# *Lactiplantibacillus argentoratensis* AGMB00912 protects weaning mice from ETEC infection and enhances gut health

**DOI:** 10.3389/fmicb.2024.1440134

**Published:** 2024-09-10

**Authors:** Ki-Nam Yoon, Jihye Yang, Seo-Joon Yeom, Sang-Su Kim, Jong-Heum Park, Beom-Seok Song, Jong-Bang Eun, Seung-Hwan Park, Ju Huck Lee, Hyeun Bum Kim, Ju-Hoon Lee, Jae-Kyung Kim

**Affiliations:** ^1^Advanced Radiation Technology Institute, Korea Atomic Energy Research Institute, Jeongeup-si, Republic of Korea; ^2^Department of Food Science and Technology, Graduate School of Chonnam National University, Gwangju, Republic of Korea; ^3^Departments of Food and Animal Biotechnology and Agricultural Biotechnology, Research Institute of Agriculture and Life Sciences, Center for Food and Bioconvergence, Seoul National University, Seoul, Republic of Korea; ^4^Korean Collection for Type Cultures, Korea Research Institute of Bioscience and Biotechnology, Jeongeup-si, Republic of Korea; ^5^Department of Animal Resources Science, Dankook University, Cheonan, Republic of Korea

**Keywords:** post-weaning diarrhea, short-chain fatty acid, *Lactiplantibacillus argentoratensis* AGMB00912, growth performance, intestinal barrier integrity, gut microbiota

## Abstract

Maintaining a healthy intestinal environment, optimal epithelial barrier integrity, and balanced gut microbiota composition are essential for the growth performance of weaning pigs. We identified *Lactiplantibacillus argentoratensis* AGMB00912 (LA) in healthy porcine feces as having antimicrobial activity against pathogens and enhanced short-chain fatty acid (SCFA) production. Herein, we assess the protective role of LA using a weaning mouse model with enterotoxigenic *Escherichia coli* (ETEC) infection. LA treatment improves feed intake and weight gain and alleviates colon shortening. Furthermore, LA inhibits intestinal damage, increases the small intestine villus height compared with the ETEC group, and enhances SCFA production. Using the Kyoto Encyclopedia of Genes and Genomes and other bioinformatic tools, including InterProScan and COGNIZER, we validated the presence of SCFA-producing pathways of LA and *Lactiplantibacillus* after whole genome sequencing. LA mitigates ETEC-induced shifts in the gut microbiota, decreasing the proportion of *Escherichia* and *Enterococcus* and increasing SCFA-producing bacteria, including *Kineothrix*, *Lachnoclostridium*, *Roseuburia*, *Lacrimispora*, *Jutongia*, and *Blautia*. Metabolic functional prediction analysis revealed enhanced functions linked to carbohydrate, amino acid, and vitamin biosynthesis, along with decreased functions associated with infectious bacterial diseases compared to the ETEC group. LA mitigates the adverse effects of ETEC infection in weaning mice, enhances growth performance and intestinal integrity, rebalances gut microbiota, and promotes beneficial metabolic functions. These findings validate the functionality of LA in a small animal model, supporting its potential application in improving the health and growth performance of weaning pigs.

## 1 Introduction

Intestinal disorders, including gut inflammation and post-weaning diarrhea, are major diseases affecting early-weaned piglets (Smith et al., 2010). Although early weaning offers the advantage of improving productivity by shortening the pig production cycle (Campbell et al., 2013), the weaning transition can be stressful for piglets due to the sudden removal from their mothers, forcing them to adapt to solid feed as milk replacement (Lallès et al., 2007; Gresse et al., 2017). These stressful environments decrease the proportion of *Lactobacillus* spp. and increase the relative abundance of *Clostridium* spp., *Provotella* spp., and Proteobacteria, including *Escherichia coli* in the intestinal microbiota of weaning piglets (Frese et al., 2015; Pajarillo et al., 2014; Mach et al., 2015; Yang et al., 2016). The loss of gut bacterial diversity may induce villous atrophy and crypt hyperplasia, which can increase intestinal permeability, making the gut more susceptible to infections by major pathogens, including *Salmonella* spp. and enterotoxigenic *Escherichia coli* (Gresse et al., 2017; Tsukahara et al., 2016; Inoue et al., 2015). Therefore, imbalances in intestinal microbiota and reduced diversity during the early stages of life can substantially increase the likelihood of intestinal disorders in weaning piglets.

*Lactiplantibacillus plantarum* has two subspecies, *L. plantarum* subsp. *plantarum* and *L. plantarum* subsp. *argentoratensis* (*L. argentoratensis*) (Bringel et al., 2005). *L. argentoratensis* is a gram-positive, bacillus-shaped, facultatively anaerobic, and facultatively heterofermentative bacterium (Kleerebezem et al., 2010). *L. argentoratensis* was first isolated from cassava, tapioca, white maize (Bringel et al., 2005) as *Lactobillcus argentoratensis*, then changed to *Lactiplantibacillus argentoratensis* by new nomenclature within its genus in 2020 (Zheng et al., 2020). Distinct and homogenous results were previously reported in a phylogenetic analysis of two genes, *recA* (encoding the recombinase A protein) and *cpn60* (encoding the GroEL chaperonin) in *L. argentoratensis* compared with *L. plantarum* subsp. *Plantarum* (Bringel et al., 2005). Accordingly, *L. argentoratensis* was deemed a new subspecies and type strain DSM 16365^T^. Another distinction between these two subspecies is the inability of *L. argentoratensis* to metabolize methyl α-D-mannoside or melezitose. In terms of facultative heterofermentative bacteria, *L. argentoratensis* can utilize homo-and heterofermentation (Kleerebezem et al., 2003). *L. argentoratensis* can ferment carbon sources via the Embden–Meyerhoff–Parnas (EMP) and phosphoketolase pathways, leading to homo-and heterofermentation, respectively (Kandler, 1983). Through the EMP and phosphoketolase pathways, *L. argentoratensis* produces pyruvate, converting it to lactate, acetate, formate, and short-chain fatty acids (SCFAs) (Quatravaux et al., 2006; Spector, 2009). During acetate production, this pathway also produces carbon dioxide and hydrogen peroxide (Christensen et al., 1999). Oxygen undergoes a Mn-dependent process to be converted to hydrogen peroxide. In this process, catalase reduces the oxygen concentration—a favorable condition for aerotolerant bacteria.

In the intestine, the gut microbiota metabolizes SCFAs such as acetate, propionate, and butyrate, which are pivotal in maintaining the intestinal health of humans and animals (Louis et al., 2007; Koh et al., 2016). SCFAs regulate the pH of the gastrointestinal tract, act as an energy source for epithelial cells within the colon, and promote epithelial cell proliferation (Koh et al., 2016; Bugaut, 1987; Comalada et al., 2006). As key mediators at the interface between the host and its microbiome, SCFAs fortify the defense against harmful microorganisms and exhibit immunomodulatory effects (Rooks and Garrett, 2016; Byndloss and Bäumler, 2018). Evidence suggests that SCFAs have a positive health impact on weaned piglets (Nakatani et al., 2018; Lingbeek et al., 2021; Qi et al., 2020). For instance, sodium butyrate reduces post-weaning diarrhea and enhances tight junction protein expression in the colonic tissues of weaning piglets (Feng et al., 2018). Notably, probiotics that produce SCFAs, such as *L. johnsonii* L531, can reduce the prevalence of harmful microbes and stabilize SCFA concentrations in weaning pigs exposed to *S. enterica* (He et al., 2019). Additionally, combining probiotics (*L. brevis, L. reuteri, L. plantarum, L. acidophilus, L. paracasei, B. licheniformis, B. subtilis, B. amyloliquefaciens, E. faecium*, and *S. cerevisiae*) reportedly has a positive influence on the gut microbiota and enhance SCFA levels in weaned piglets (Lu et al., 2018; Oh et al., 2021; Wang et al., 2023).

In this study, we assess the effects of supplementing the diets of weaning mice with *Lactiplantibacillus argentoratensis* AGMB00912 (LA) isolated from the feces of healthy pigs on growth performance. We hypothesized that supplementation with LA would enhance growth performance in the weaning phase, potentially through SCFA production, improve intestinal integrity, and increase intestinal microbiota diversity. Additionally, *in vitro* tests for SCFAs, including lactate, acetate, and butyrate, were performed. Our results show that LA exhibits antibacterial activity against microorganisms associated with post-weaning diarrhea in weaning piglets. We further evaluated the protective role of LA in an enterotoxigenic *E. coli* infection model using weaning mice to preliminarily assess the functionality and protective effects of LA before conducting validation experiments in pigs.

## 2 Methods

### 2.1 Pathogen strains and preparation

Enterotoxigenic *E. coli* (ETEC) Korea Veterinary Culture Collection (KVCC) 0548 (K88ac), ETEC KVCC 0549 (K88ab), ETEC KVCC 0546 (K99), Shiga toxin-producing *E. coli* (STEC) KVCC 0544 (STa/987p), STEC KVCC 1423 (STa/LT/Stx2e), STEC KVCC 1424 (Stx2e), and *Salmonella Typhimurium* (ST454) were acquired from KVCC (Anyang, South Korea) and stored at −80°C using 20% (v/v) glycerol (Sigma-Aldrich, Germany). The strains were activated in Luria–Bertani (LB) broth (BD, USA) and incubated at 37°C for 24 h.

### 2.2 Isolation and identification of gut bacteria from swine fecal samples

Stool samples from healthy pigs were obtained from the National Institute of Animal Science (Cheonan, South Korea). A total of 10 stool samples were used, and they were stored at −80°C until bacterial isolation. Fecal samples were suspended in sterilized phosphate-buffered saline (PBS) solution and serially diluted to a factor of 10^−6^. The resulting diluted specimens were cultured on tryptic soy agar supplemented with 5% sterile sheep blood. The culture was incubated within an anaerobic chamber (Coy Laboratory Product, USA), maintaining an environment of 90% N_2_, 5% CO_2_, and 5% H_2_ at 37°C over 3 d. Following incubation, single colonies were isolated and streaked onto fresh plates containing tryptic soy agar supplemented with 5% sterile sheep blood for further analysis. In total, 100 different bacterial strains were obtained, including 10 strains of lactobacilli. Isolated gut bacteria (AGMB) were identified by sequencing the 16S rRNA gene using a universal primer set (27F/1492R).

### 2.3 Cell-free supernatant preparation and antimicrobial activity screening

Each identified microorganism was inoculated into reinforced clostridial medium (MBcell, South Korea) broth and incubated in an anaerobic chamber at 37°C for 48 h (Coy Laboratory Product). The culture was centrifuged at 3,264 × *g* for 20 min at 4°C. Subsequently, the cell-free supernatant (CFS) was collected and sterilized using a 0.2 μm syringe filter (Millipore, USA). An agar well-diffusion assay was conducted to screen the antimicrobial activity of AGMB CFS using a previously published protocol (Divyashree et al., 2022) with slight modifications. Briefly, ETEC, STEC, and ST454 strains were cultivated in LB broth at 37°C for 24 h (10^8^ CFU/mL), and 1 mL of culture was inoculated into 100 mL of LB Agar (BD, USA) before being distributed onto Petri dishes. Subsequently, the inoculated LB agar was punctured within the wells using a sterile corkborer with an 8 mm diameter. Next, 100 μL of CFS was deposited into the wells and incubated at 37°C for 24 h. The diameters of the inhibition zones were quantified. Kanamycin (25 μg/mL) was used as a positive control, and the CFSs of LA were selected due to their antibacterial activity. For the selective cultivation of LA, the antibacterial activity of LA was conducted after culturing LA in De Man-Rogosa-Sharpe (MRS) broth (BD Difco^™^, USA) under anaerobic conditions for 24 h.

### 2.4 Antimicrobial activity of LA

#### 2.4.1 96-well broth culture assay

The antimicrobial efficacy of the CFS in LA was assessed using a 96-well microtiter plate in accordance with the procedure outlined by Mlambo et al. (2022) with slight modifications. First, 20 μL of CFS was incorporated into each well containing 180 μL of the pathogen strain (10^6^ CFU/mL). Then, antimicrobial evaluations were made using kanamycin (25 μg/mL) and MRS broth as positive and negative controls, respectively. Absorbance readings for each well were taken at 600 nm at 0, 4, and 12 h post-incubation at 37°C.

#### 2.4.2 Co-culture assay

Furthermore, co-cultures of the LA strain and pathogens were evaluated using the co-culture method described by Wang J. et al. (2018), with slight modifications. After incubation, LA and the pathogens (ETEC, STEC, and ST454) were adjusted to a concentration of 10^6^ CFU/mL in sterile MRS broth. LA and the pathogens were then collectively inoculated into fresh MRS medium, reaching a final volume of 10 mL at a concentration of 10^6^ CFU/mL. Following incubation, 1 mL of monoculture samples and 100 μL of co-culture samples were harvested at 0, 6, 12, and 24-h intervals to evaluate bacterial proliferation. The samples were then diluted and plated, with LA on MRS agar and pathogens (ETEC, STEC, and ST454) on LB agar, and incubated at 37°C to allow for colony growth.

#### 2.4.3 Assessment of CFS inhibitory effect

The CFS inhibitory effect of LA was evaluated by heat and enzyme treatment and pH adjustment, as described by Mariam et al. (2014), with slight modifications. A broth culture assay using 96-well plates was performed on heat- (60°C for 15 min, 98°C for 15 min, and 121°C for 15 min) and enzyme-treated (α-amylase, catalase, proteinase-K, papain, pepsin, trypsin, and chymotrypsin) CFSs with pH adjusted to 6.8–7.0, following the protocol described in Section 2.4.1.

### 2.5 Whole-genome sequencing and bioinformatic analysis

#### 2.5.1 Genomic DNA extraction

Genomic DNA extraction was conducted using 1 mL of exponentially growing LA culture (OD_600_ = 0.8 ~ 1.0) with the G-spin^™^ Genomic DNA Extraction Kit (Intron Biotechnology, South Korea) according to the manufacturer’s instructions. For the subsequent genome sequencing step, quality control was performed according to the manufacturer protocol recommended by Oxford Nanopore Technologies (ONT).

#### 2.5.2 Genome sequencing and annotation

Before whole-genome sequencing of LA, the extracted genomic DNA was prepared following the protocol of the genomic sequencing kit SQK-LSK109 (Oxford Nanopore Technologies (ONT), UK). The prepared library was loaded onto a primed R9.4 Spot-On Flow cell (FLO-MIN106; ONT). Sequencing was performed using a MinION Mk1B sequencer. The sequenced FAST5 files were base-called using the Oxford Nanopore software MinKNOW (v.22.12.7) with parameters for the library type. After genome sequencing, qualified raw sequence reads were used for genome assembly using Trycycler (v.0.5.3) (Wick et al., 2021) and predicted and annotated using Prokka (v.1.14.6) (Seemann, 2014). Functional analysis of predicted open-reading frames was performed using InterProScan (v. 5.64–96.0) (Jones et al., 2014). Clusters of Orthologous Genes functional categorization and metabolic pathway analysis were conducted using COGNIZER and Kyoto Encyclopedia for Genes and Genomes (KEGG) Automatic Annotation Server (KAAS), respectively (Jones et al., 2014; Bose et al., 2015). Genome maps were visualized using Artemis (v.16.0.17) (Carver et al., 2012) (Wellcome Sanger Institute, UK) and GenVision Pro (v.17.4) (DNASTAR, USA).

#### 2.5.3 Comparative genome analysis

For taxonomic identification based on comparative analysis, the average nucleotide identity (ANI) values between *Lactiplantibacillus* strains were evaluated using FastANI (v.1.32) (Supplementary Table S1; Jain et al., 2018). Constructed based on calculated distance values, the phylogenetic tree utilizing ANI was visualized using RStudio software within the R package (v.4.2.1) (R Core Team, 2022). For the pan-genome analysis, LA and nine other *L. argentoratensis* strains with genomic features obtained from the National Center for Biotechnology Information (NCBI) were compared using Roary (v.3.11.2) (Supplementary Table S2; Page et al., 2015). The pan-genome analysis was visualized using Phandango (v.1.3.0) (Hadfield et al., 2018) and RStudio software.

### 2.6 Prediction of virulence factors and antibiotic resistance genes

To assess safety of LA at genomic level, virulence and pathogenicity factors were predicted using a virulence factor database (Chen et al., 2005). In addition, antibiotic resistance and the possibility of horizontal gene transfer (HGT) events were determined using the Resistance Gene Identifier (v6.0.2), based on the Comprehensive Antibiotic Resistance Database (v.3.2.7) (CARD) (Alcock et al., 2023) and HGTphyloDetect (Yuan et al., 2023).

### 2.7 *In vitro* safety assessment

#### 2.7.1 Antibiotics resistance analysis

An overnight culture of LA was inoculated at a ratio of 1% (w/w) into 0.6% (w/v) MRS soft agar supplemented with 0.05% (w/v) L-cysteine hydrochloride. The inoculated soft agar was poured onto a base agar plate and allowed to solidify at room temperature. Each ETEST^®^ (bioMérieux, France) strip of antibiotics was then placed onto the plate, which was incubated at 37°C for 12 h. Following incubation, the inhibition zone was observed and the minimum inhibitory concentration (MIC) was determined. The analysis was conducted in duplicate, and the highest MIC value from the two experiments was considered the final MIC. Cut-off value was referred by European Food Safety Authority’s (EFSA) (Likotrafiti et al., 2018).

#### 2.7.2 Cytotoxicity test

Caco-2, a human colon carcinoma cell line, was cultured in DMEM media (Welgene, South Korea) supplemented with 10% fetal bovine serum (Welgene) and 1X penicillin–streptomycin (Welgene). The cells were maintained in a humidified atmosphere of 5% CO_2_ and 95% air at 37°C. Upon reaching 90% confluency, the cells were seeded into 12-well plates at a density of 5 × 10^4^ cells/well (SPL, South Korea). The culture medium was replaced every other day, and the cells were differentiated over a period of 10 days. After differentiation, bacterial cells were added to each well at concentrations of 10^7^, 10^8^, and 10^9^ CFU/well in DMEM media containing 10% fetal bovine serum, followed by a 24-h incubation under standard conditions. The cytotoxicity was assessed using lactate dehydrogenase (LDH) assay (EZ-LDH, DoGenBio, South Korea) conducted using the cell supernatant according to the manufacturer’s instructions. Basal media-treated cells served as the negative control, while *E. coli* O157 ATCC 48395 was used as the positive cytotoxic control strain.

#### 2.7.3 Hemolysis assay

To assess hemolytic activity of bacteria, AGMB00912 and control strains were streaked onto tryptic soy agar (BD Difco, USA) supplemented with 5% (v/v) defibrinated sheep blood (KisanBio, South Korea). The plates were incubated at 37°C for 12 h to observe its hemolytic activity. Control strains were *Staphylococcus salivarius* ATCC 19250, *S. aureus* ATCC 29213, and *S. epidermis* ATCC 35983 for alpha-hemolysis (greenish halo), beta-hemolysis (clear halo), and gamma-hemolysis (no halo), respectively.

### 2.8 Animal experiment design and sample preparation

All procedures involving animals were conducted with strict adherence to ethical guidelines under the approval of the Korea Atomic Energy Research Institute’s Institutional Animal Care & Use Committee (approval no. KAERI-IACUC-2022-011). The animals were acclimatized for 1 week before experimental grouping and monitoring. All efforts were made to minimize suffering throughout the study, including euthanasia via cervical dislocation, in line with American Veterinary Medical Association guidelines. This study’s commitment to animal welfare reflects our dedication to ethical research principles.

ICR mice (male), aged 2 weeks, were acquired from GBIO Inc. (South Korea) and were kept with their mother, nursing on maternal milk, during a one-week acclimatization period. The environment was controlled at a temperature of 22 ± 2°C and relative humidity of 24 ± 2%, under a 12-h light/dark cycle. After the acclimatization period, at 3 weeks of age, the mice were separated from their mother and randomly assigned to four groups. Initially, survival rates were assessed using 10 mice per group. In the EC group, deaths occurred after day 12, with a 50% mortality rate observed by day 14. Based on these findings, the feeding experiment was conducted over 12 days with 5 mice per group (*n* = 5 for each group). The bacteria were administered via oral gavage. (1) Control group: daily dose of 100 μL of 1X PBS from day 0 to day 12; (2) EC group: 100 μL of 1X PBS daily from day 1 to day 8, and 100 μL of ETEC (10^9^ CFU/mL in 1X PBS) on days 9 and 11; (3) LA group: 100 μL of *Lactiplantibacillus argentoratensis* AGMB00912 (10^9^ CFU/mL in 1X PBS) orally daily from day 0 to day 12; and (4) LA pretreatment (LE) group: 100 μL of LA daily from day 1 to day 8 and 100 μL of ETEC (10^9^ CFU/mL in 1X PBS) on days 9 and 11. The LA dose was selected based on other studies that have demonstrated the efficacy and safety of similar probiotic dosages in animal models (He et al., 2019; Taverniti et al., 2021).

The feeding experiment was conducted over 12 d. Throughout the study, the body weight and feed intake of weaning mice were measured daily. Additionally, we assessed the variations in body weight percentage, cumulative weight gain, and feed-to-gain ratio. After the feeding study, the mice were sacrificed by cervical dislocation and subsequently dissected. We collected blood samples using cardiac puncture and centrifuged them at 3,264 × *g* at 4°C for 20 min to facilitate serum collection. Colon length was measured, and the medial segments of the duodenum, jejunum, ileum, and colon were stabilized with a 10% (v/v) solution of neutral buffered formalin (Sigma-Aldrich) for hematoxylin and eosin staining. Fecal specimens were stored at −80°C until further analyses of SCFAs and the 16S rRNA gene were performed.

### 2.9 Blood hematological profile, cytokine, and antioxidant analysis

Analysis of the hematological profile, including white blood cell, red blood cell, and hemoglobin (Hb) counts in whole blood, was conducted using a Drew Hemavet 950FS analyzer (Drew Scientific, Oxford, CT, USA) following the manufacturer’s protocols. The quantification of serum tumor necrosis factor (TNF)-α and interleukin (IL)-6 was executed using a mouse-designated enzyme-linked immunosorbent assay (ELISA) kit (R&D Systems, USA), according to the manufacturer’s instructions, the detection limits were 31.2–2,000 pg./mL for TNF-α and 15.6–1,000 pg/mL for IL-6. Serum antioxidant metrics, including catalase (Catalog No. EIACATC), superoxide dismutase (Catalog No. EIASODC), and glutathione (Catalog No. EIAGSHC), were assessed using commercial kits from Invitrogen (Thermo Fisher Scientific, Waltham, MA, USA), according to the manufacturer’s instructions.

### 2.10 Histology analysis

Tissues from the small intestine and colon, fixed with 10% neutral buffered formalin, were integrated into a paraffin medium. Post-dehydration, 4-μm-thick sections were subjected to staining using hematoxylin and eosin, enabling subsequent histological investigations. Images were acquired using a slide scanner (Carl Zeiss AXIO Z1; Carl Zeiss, Germany), and ZEN software (Zeiss, Jena, Germany) was used to determine villus height and crypt depth within the intestine. A subsequent calculation yielded the villus height-to-crypt depth ratio.

### 2.11 Fecal SCFA determination using gas chromatography/mass spectrometry

Analysis of SCFAs, including acetic, propionic, isobutyric, butyric, isovaleric, and valeric acids, was conducted following the procedure described by Zhang et al. (2019) with slight adjustments (Zhang et al., 2019). Fecal samples (30 mg) were blended with 300 μL of high-performance liquid chromatography quality water using a Bead Ruptor, operating at 8,606 × g for 20 s, then subjected to agitation at 4°C for 30 min and centrifuged at 13,000 × *g* and 4°C for another 30 min. Next, 100 μL of the supernatant was allocated to a fresh tube, acidulated with 10 μL of 5 M HCl, and subjected to extraction with 100 μL of anhydrous diethyl ether, proceeding through a sequence of vortexing, cooling, and centrifugation (10,000 × *g*, 5 min, 4°C). The SCFA-containing ether layer was transferred to a new tube with Na_2_SO_4_, repeated twice, centrifuged (10,000 × *g*, 2 min, 4°C), and 100 μL of the final solution was transferred to a gas chromatography vial for derivatization with 5 μL of N, O-bis (trimethylsilyl) trifluoroacetamide, vortexed, and incubated at 70°C for 40 min and 37°C for another 40 min. The derivatized SCFAs were analyzed using a gas chromatography/mass spectrometry analyzer (QP 2010 Ultra; Shimadzu Corporation) equipped with a DB-5MS UI capillary column under the specified temperature and voltage conditions. A 1-μL sample was injected in a split mode 10:1 with helium as a carrier gas. The temperature protocol and electron impact mode for the ion source were set as previously detailed, and SCFAs were identified and quantified by comparing the target and confirmed ions using the selected ion monitoring mode against external standards in a 20-min run, collecting mass spectral data from a mass range of 40–400 m/z. The SCFA concentration was calculated after confirming the retention time and mass spectrum using external standards.

### 2.12 16S rRNA gene sequencing analysis

Total DNA was extracted from 200 mg of collected feces per sample using a QIAamp Fast DNA Stool Mini Kit (Qiagen, Germany), according to the manufacturer’s instructions. DNA concentrations were measured using a Colibri Microvolume Spectrometer (Titertek-Berthold, Germany), and only DNA samples with an OD_260/280_ ratio between 1.85 and 2.15 were qualified for use in further analyses. 16S rRNA amplicons were prepared with a V3–V4 targeting universal primer set (799F-mod6/1114R) under the following PCR conditions: one cycle of 98°C for 3 min, 30 cycles of 98°C for 10 s, 57°C for 5 s, and 68°C for 1 s, and one cycle of 72°C for 5 min. After 16S rRNA PCR, the amplicons were purified using Wizard^®^ SV Gel and PCR Clean-Up System (Promega, USA). NGS was conducted using an Illumina MiSeq System at CancerRop (South Korea).

### 2.13 Bioinformatic analysis

The sequences of each amplicon sequence variant were analyzed using BLAST+ (v2.9.0) and the NCBI 16S rRNA database to assign taxonomic information based on the highest similarity with the subjects in the database. The taxonomy assignment was discarded if the best hit from the database showed a query coverage or identity of <85%. For multiple alignments of amplicon sequence variant sequences, the MAFFT (v7.475) program was used, and a phylogenetic tree was generated using the FastTreeMP (v2.1.10) program. Using the abundance and taxonomic information of the amplicon sequence variants, various microbial community comparative analyses were conducted utilizing QIIME2 (v1.9). To ascertain species diversity and evenness within the microbial communities of the samples, the Shannon and Inverse Simpson indices were calculated. Alpha diversity was verified using rarefaction curves and Chao1 values. Using weighted and unweighted UniFrac distances as a basis, beta diversity was quantified within comparative groups to probe variations in the microbial community among samples. Visualization of the interrelationships between samples was achieved using principal coordinate analysis (PCoA) and the Unweighted Pair Group Method with Arithmetic mean tree. To identify specific biomarkers distinguishing EC from other groups, linear discriminant analysis effect size (LEfSe) was executed using the microbiomeMarker (v1.2.1) R package. Linear discriminant analysis (LDA) scores that ranked differential taxa from the phylum to the genus level were projected onto a cladogram, visualized with GraPhlAn (v1.1.3, Python v2.7), and the differential species level was displayed as a histogram (Prism, v8.0.1). Metabolic functional pathways were predicted using a phylogenetic investigation of communities by reconstruction of unobserved states (PICRUSt2) pipeline (v2.5.2). The assignment of metabolic functional pathways adhered to the KEGG Ortholog database and the KEGG abundance data for all predicted pathways were recalibrated to relative abundance (%).

### 2.14 Statistical analysis

Statistical analyses were performed using GraphPad Prism (v8.0.1) (GraphPad Software, Inc., San Diego, CA). The antimicrobial activity of LA’s CFS, the evaluation of factors inhibiting its antimicrobial activity, and the analysis of organic acid content were assessed using one-way ANOVA followed by Tukey’s multiple comparisons test. The results are presented as the mean ± SD (*n* = 3), with significance set at *p* < 0.05. ETEC infection data in weaning mice were expressed as the mean ± SD (*n* = 5). Differences between the EC and other groups (Con, LA, and LE) were analyzed using an unpaired t-test. Gut microbiota diversity was analyzed using QIIME2 (v1.9). Alpha diversity was assessed using Chao, Shannon, and Simpson diversity indices, and group differences were evaluated using the Kruskal–Wallis test. Beta diversity significance was determined using both unweighted and weighted UniFrac distances. The significance of beta diversity was assessed using permutational multivariate analysis of variance (PERMANOVA) with the vegan package (version 2.6–4) in R. The LEfSe analysis employed an LDA effect size cut-off of ≥3 and an alpha of 0.05 for the initial Kruskal–Wallis sum-rank test, followed by the Wilcoxon rank-sum test. Core microbiota analysis was performed using default parameters with 20% sample prevalence and 0.2% relative abundance. Differences in the predicted metagenomic functions between the groups were analyzed using the R software package ALDEx2, with significance set at *p* < 0.05.

### 2.15 Nucleotide sequence accession number

The genome sequence of *L. argentoratensis* AGMB00912 is available in the GenBank database under the accession number CP136431.

## 3 Results

### 3.1 LA exhibits antimicrobial activity against porcine pathogenic strains through SCFA production

**Figure 1:**
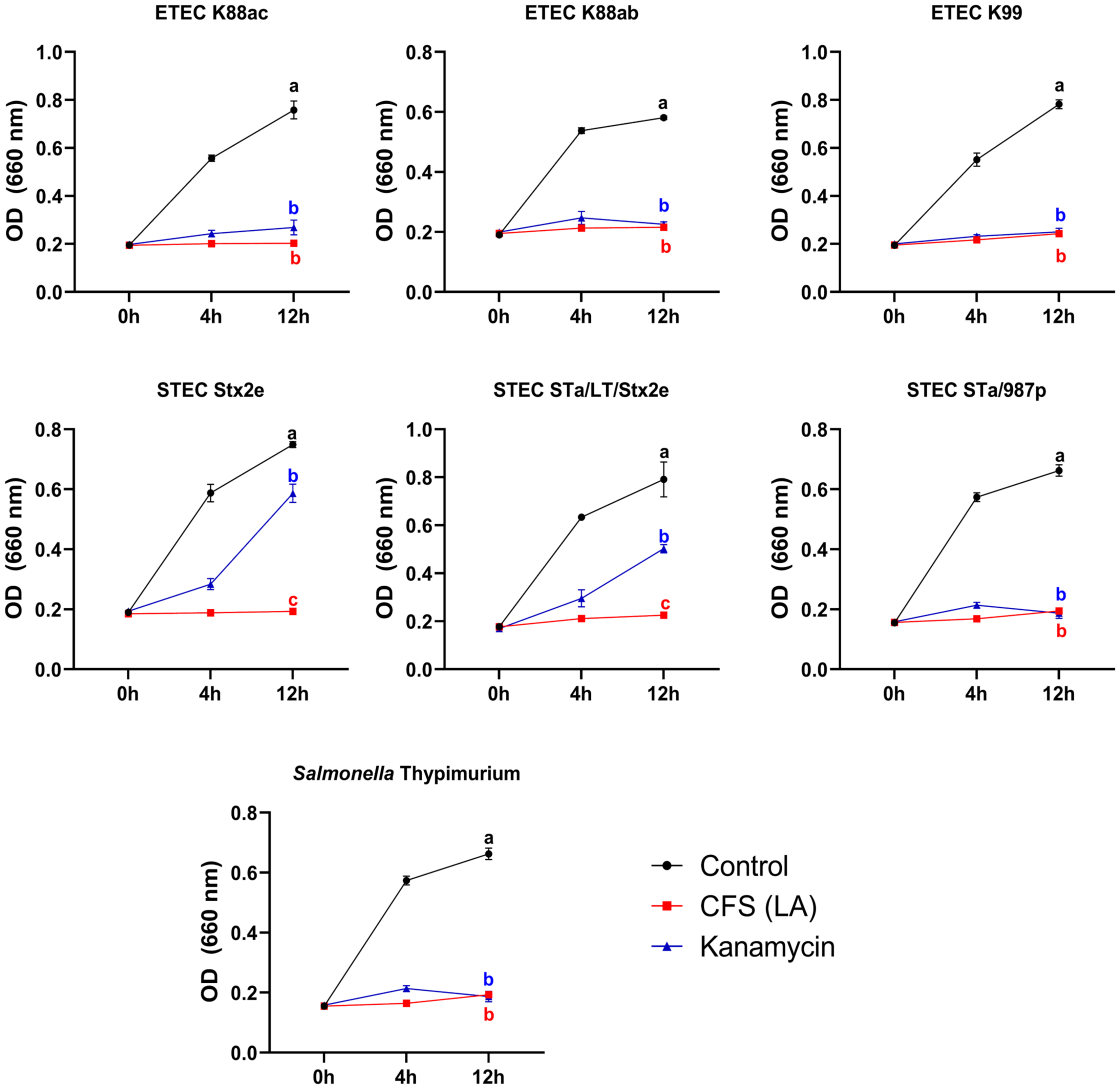
Growth analysis of pathogenic bacteria. Inhibition of intestinal pathogenic bacteria growth by AGMB00912 examined by adding 10% LA cell-free supernatant (CFS) to enterotoxigenic *Escherichia coli* (ETEC K88ac, K88ab, K99), Shiga toxin-producing *Escherichia coli* (STEC Stx2e, STa/LT/Stx2e, STa/987p), or *Salmonella* Typhimurium (ST454) (10^7^ CFU) and measuring growth over 0, 4, and 12 h. Data represent the mean ± SD (*n* = 3). Statistical analysis was performed using one-way ANOVA followed by Tukey’s multiple comparisons test. Different letters (a, b, c) indicate significant differences between groups (*p* < 0.05).

Among the CFSs of the 100 isolated gut bacterial strains, LA showed a clearing zone (> 10 mm) against ETEC, STEC, and ST454 (Supplementary Table S3 and Supplementary Figure S1). Additionally, the 96-well broth assay results demonstrated that the CFS of LA exhibited inhibitory activity against pathogenic strains (Figure 1). Notably, we observed that the antibacterial activity of kanamycin was less effective against the STEC (STa/LT/Stx2e, Stx2e) strain, whereas the LA’s CFS significantly inhibited STEC growth. Furthermore, we investigated the antimicrobial activity of LA against pathogens under mono-and co-culture conditions (Supplementary Figure S2). Mono culture conditions, ETEC (K88ac, K88ab, and K99), STEC (STa/987p, STa/LT/Stx2e, and Stx2e), and ST454 were cultured in MRS broth at 10^8^ CFU/mL for 24 h. In contrast, in a co-culture with LA, the growth of these pathogens was inhibited, with viable counts remaining constant until 12 h, followed by a rapid decline, becoming undetectable at 24 h.

**Figure 2:**
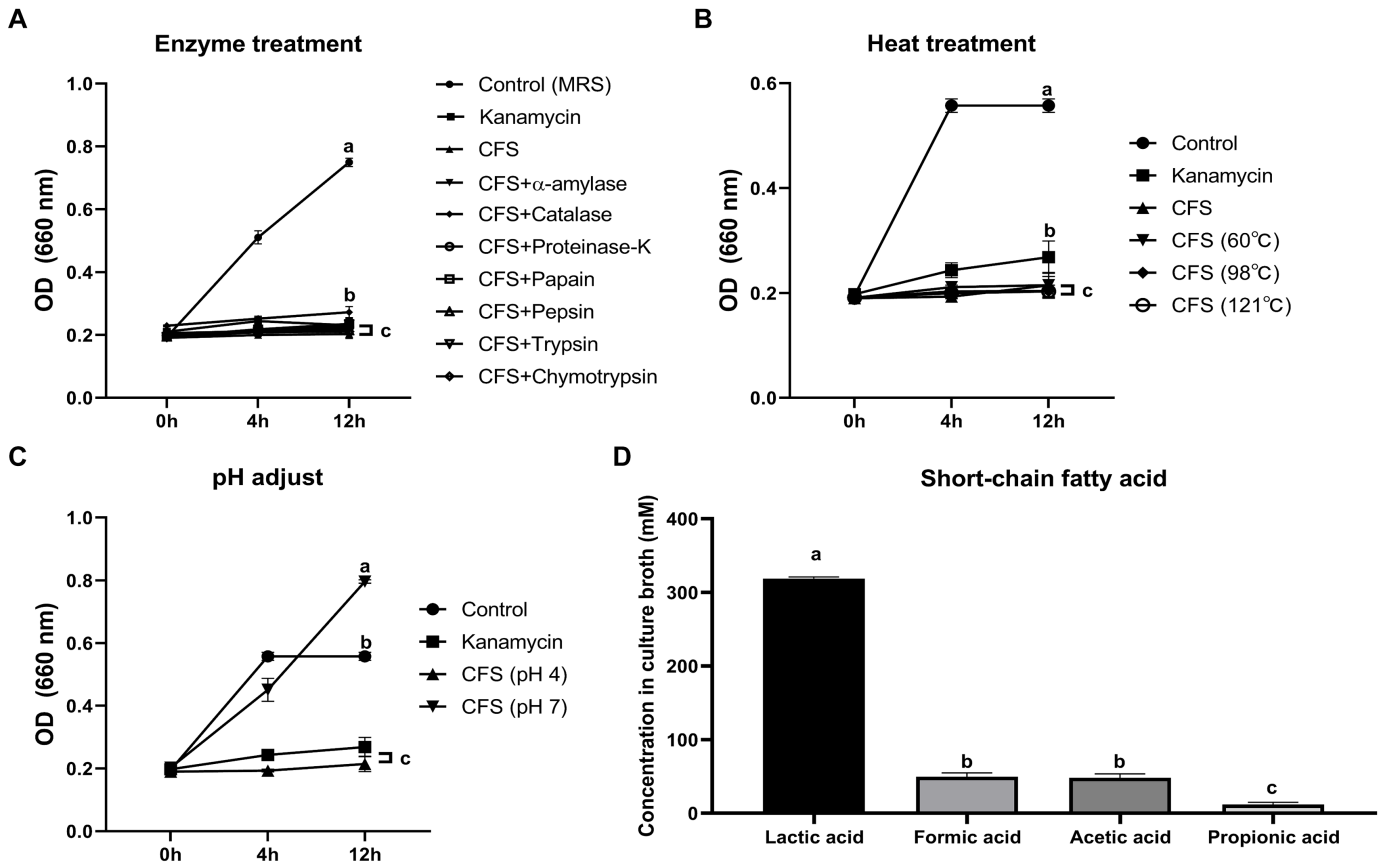
Evaluation of enzyme **(A)**, heat **(B)**, and pH **(C)** effects on antimicrobial activity against ETEC K88ac of AGMB00912 CFS. Short-chain fatty acid (SCFA) content of AGMB00912 CFS **(D)**. Data represent the mean ± SD (*n* = 3). Statistical significance was determined by one-way ANOVA followed by Tukey’s multiple comparisons test. Different letters (a, b, c) indicate statistically significant differences between groups (*p* < 0.05).

To elucidate the mechanism by which the LA’s CFS exhibited antibacterial activity, we conducted inhibitory activity tests (Figure 2). The antimicrobial activity against ETEC was lost when the pH level of the CFS was modulated to 6.8, whereas neither heat nor enzyme treatment inhibited ETEC growth. Consequently, we analyzed the SCFA content in the LA CFS using high-performance liquid chromatography and observed the abundance of lactic, formic, propionic, and acetic acids (Figure 2D). These results suggest that LA’s antibacterial activity can be attributed to the decrease in pH, an outcome of SCFA production.

### 3.2 Genetic characteristics of LA supports its antibacterial activity

#### 3.2.1 General genomic characteristics of LA

**Figure 3:**
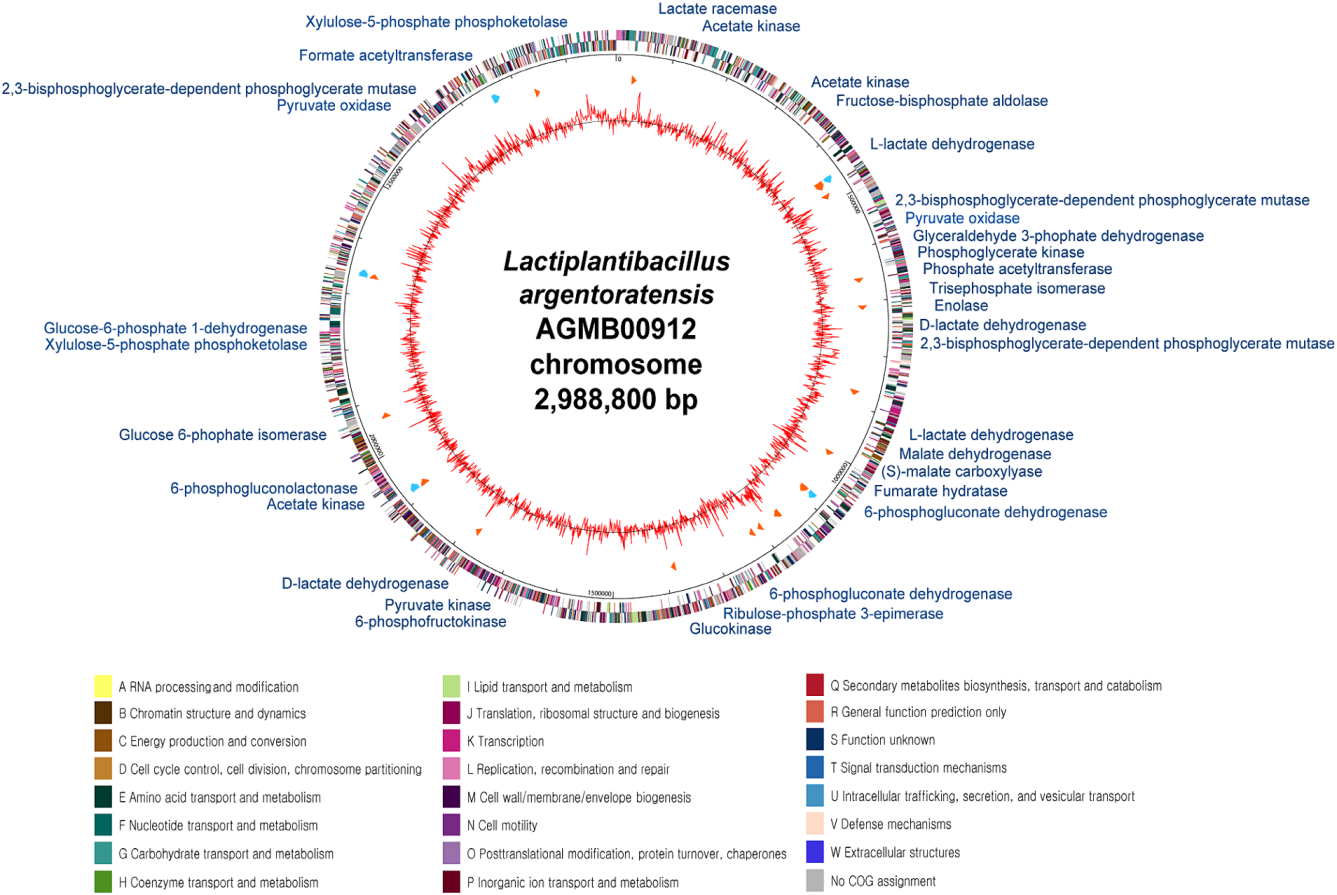
Circular genome map of *L. argentoratensis* AGMB00912. The outer circle indicates the location of all annotated open-reading frames (ORFs) in double strands colored by clusters of orthologous groups (COG), and the inner circle with red peaks indicates GC contents. Sky blue arrows (marked between these circles) indicate rRNA operons, and orange arrows indicate tRNAs. The color coding of COG categories is indicated. The annotated functional genes with blue represent genes associated with organic acid production.

**Figure 4:**
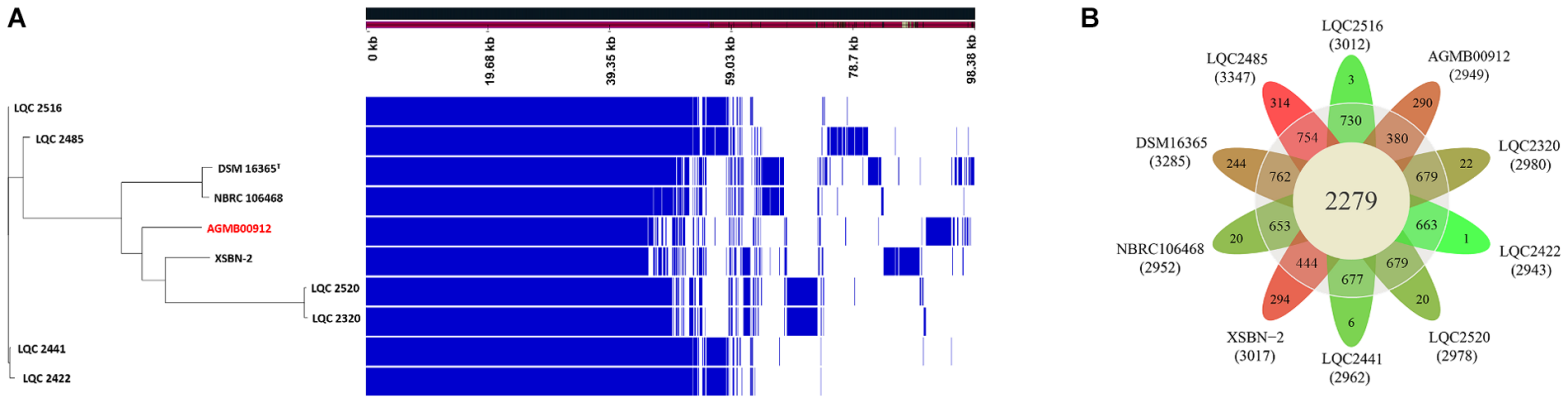
Pan-genome analysis ofAGMB00912 and nine other *L. argentoratensis* strains. **(A)** Ratio and size plot of core-and pan-genome generated using Roary 3.11.2 and visualized with Phandango 1.3.0. Each row corresponds to one strain; each column represents an orthologous gene family. **(B)** Flower plot of the pan-genome showing the core, accessory, and strain-specific genes. The number of core and accessory genes are displayed in the inner circle (yellow) and outer circle (gray), respectively. The number of strain-specific genes is shown in the petals of each strain. The number under the strain name denotes the total genes in the genome of each strain. The petal color gradient indicates the lowest strain-specific gene number in green and the highest strain-specific gene number in red.

General genome features of the LA are summarized in Supplementary Table S1 and Figure 3. The LA genome is 2,988,800 bp double-stranded circular DNA with a GC content of 45.43%, comprising 2,869 protein-coding DNA sequences, 64 tRNA genes, and 16 rRNA genes. For comparative genome analyses of LA, phylogenetic ANI tree and pan-genome analysis were conducted. The ANI tree of the 20 *Lactiplantibacillus* strains, including *L. argentoratensis, L. pentosus, L paraplantarum,* and *L. plantarum* subsp. *plantarum* was clustered into four groups depending on their species (Cluster I*, L. pentosus*; Cluster II, *L. paraplantarum*; Cluster III, *L. plantarum* subsp. *plantarum*; and Cluster IV, *L. argentoratensis*) (Supplementary Figure S3). Cluster IV showed the smallest distance from Cluster III, further confirming that *L. argentoratensis* diverged from *L. plantarum* subsp. *plantarum* (Bringel et al., 2005). LA was included in the *L. argentoratensis* cluster and was closest to *L. argentoratensis* LQC 2422 (Accession No. GCA018551525), with 98.51% shared identity, suggesting the LA was identified as *L. argentoratensis*. Pan-genome analysis of LA and the other nine *L. argentoratensis* strains documented in NCBI revealed that the pan-genome comprised 4,917 genes (Figure 4). Of these, 2,279 genes were identified as core genes shared among all strains, whereas 1,424 were accessory genes shared by two or more strains but not by all. Furthermore, 1,214 genes were specific to individual strains, with 290 unique to LA (Figure 4B).

#### 3.2.2 Predicted fermentation capability of LA

**Figure 5:**
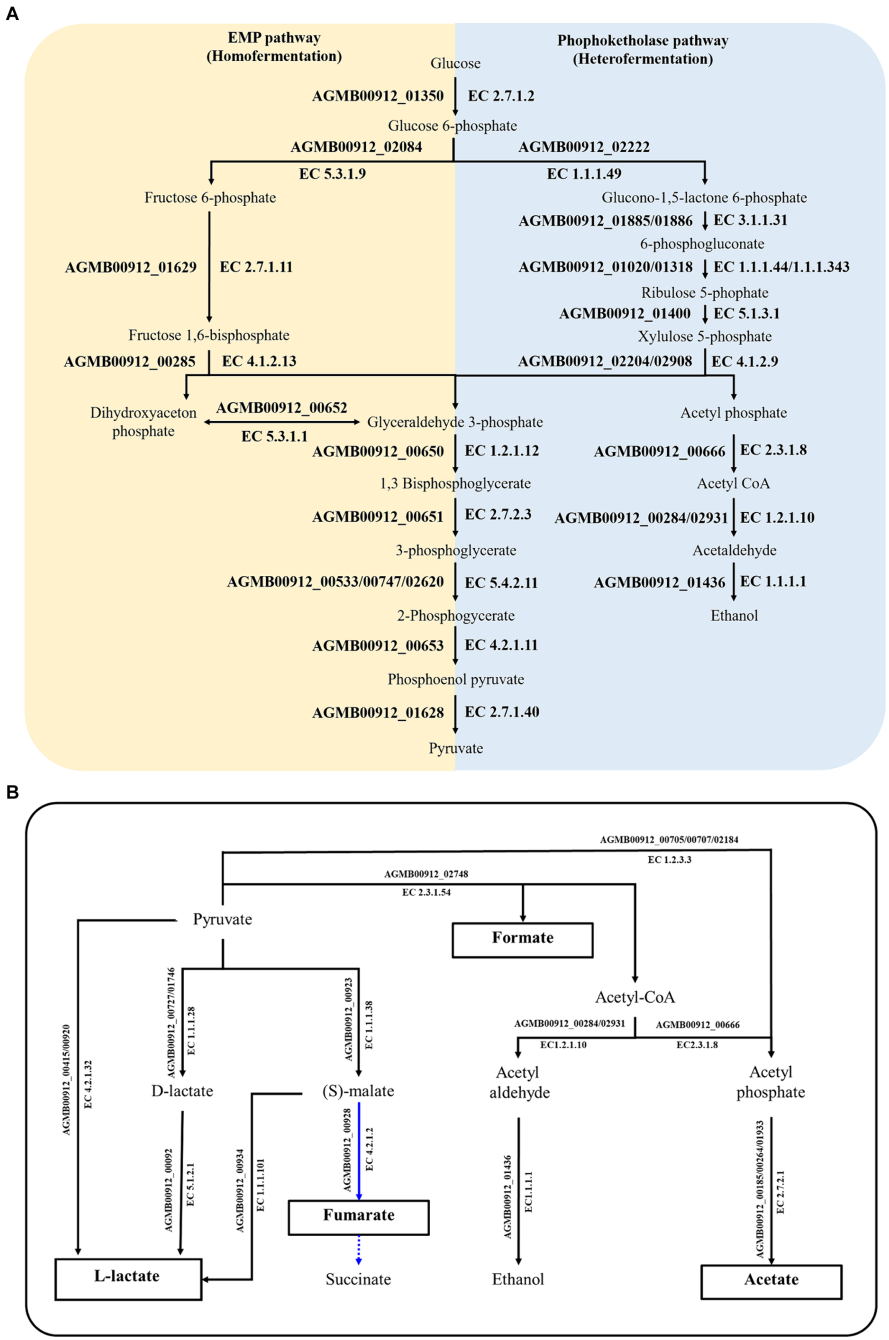
Summary of genetically predicted pathways for producing organic acid in AGMB00912. **(A)** Predicted fermentation pathways in AGMB00912. The yellow and blue areas denote homo-and heterofermentation, respectively. **(B)** Predicted SCFA-producing pathways in AGMB00912. The organic acids in white boxes represent the products that could be generated by AGMB00912. Solid lines represent the potential pathways utilized by AGMB00912; dashed lines represent absent pathways. Blue lines represent the reductive branch of the TCA cycle under anaerobic conditions.

Considering that LA showed antimicrobial activity against intestinal pathogenic bacteria (Supplementary Figure S2), it was posited that LA lowers pH by producing SCFAs or other organic acids (Cherrington et al., 1991; Ricke, 2003; Sun and O'Riordan, 2013; Broom, 2015). LA likely utilizes homo-and heterofermentation to produce lactate, acetate, succinate, and formate (Wang et al., 2021). As a result of confirming this possibility via KEGG analysis, LA was found to produce lactate, acetate, and formate via the EMP pathway (i.e., homofermentation) and the phosphoketolase pathway (i.e., heterofermentation) (Supplementary Table S4; Figures 3, 5A). Both pathways are involved in converting glucose to pyruvate; however, they follow different routes. In the EMP pathway, fructose-1,6-bisphosphate stems from the conversion of glucose; it subsequently undergoes a split into dihydroxyacetone phosphate and glyceraldehyde 3-phosphate. In contrast, in the phosphoketolase pathway, glucose is converted into xylulose-5-phosphate, which is further split into acetyl phosphate and glyceraldehyde 3-phosphate. In the EMP pathway, dihydroxyacetone phosphate molecules are eventually transformed into glyceraldehyde 3-phosphate and ultimately converted to lactate, resulting in homolactic fermentation. Meanwhile, acetyl phosphate is transformed into ethanol via the phosphoketolase pathway, leading to a heterofermentation process.

The produced pyruvate was converted to lactate by L-lactate dehydrogenase (AGMB00912_00415 and AGMB00912_00920; EC 4.2.1.32) (Figure 5B). LA can also produce D-lactate and then convert it to L-lactate, operating sequentially with D-lactate dehydrogenase (AGMB00912_00727 and AGMB00912_01746; EC 1.1.1.28) and lactate racemase (AGMB00912_00092; EC 5.1.2.1). Moreover, LA produces L-lactate from oxaloacetate via (S)-malate using malate dehydrogenase (AGMB00912_00923; EC 1.1.1.38) and (S)-malate carboxylase (AGMB00912_00934; EC 4.1.1.101). LA also generates acetate from acetyl phosphate using acetate kinase (AGMB00912_00185, AGMB00912_00264, and AGMB00912_01933; EC 2.7.2.1). To produce acetyl phosphate, LA uses acetyl-coenzyme A (CoA) with phosphate acetyltransferase (AGMB00912_00666; EC 2.3.1.8) or pyruvate with pyruvate oxidase (AGMB00912_00705, AGMB00912_00707, and AGMB00912_02184; EC 1.2.3.3). For formate production, LA operates the pathway from pyruvate to acetyl-CoA using formate C-acetyltransferase (AGMB00912_02748; EC 2.3.1.54). In a previous study, lactic acid bacteria were shown to generate succinate by converting malate to fumarate in the reductive branch of the tricarboxylic acid cycle under anaerobic conditions (Wang et al., 2021). However, LA can only convert malate to fumarate and not succinate using malate dehydrogenase (AGMB00912_00923; EC 1.1.1.38) and fumarate hydratase (AGMB00912_00928; EC 4.2.1.2). Given that fumarate is also a weak acid, its production could impact the pH-lowering effect of LA.

Considering the quantification results of organic acids in the LA CFS (Figure 2D), this KEGG analysis supports the SCFA production of LA, especially lactate, formate, and acetate. Furthermore, the metabolic pathway associated with organic acid production was observed in all other *L. argentoratensis* strains in the pan-genome analysis, suggesting that the results of this study could explain the representative characteristics of *L. argentoratensis* (Supplementary Table 5).

### 3.3 Bioinformatics and *in vitro* safety assessment of LA

To ensure the safety of LA, two approaches, bioinformatics analysis and *in vitro* assessement, were conducted. First, virulence factor database analysis was performed to identify virulence factors within the genome. Even when less stringent criteria (>60% identity, >60% coverage, and E-value <1e-10) were used, no potential virulence factors were identified. In the CARD analysis for the detection of antibiotic resistance genes, *L. argentoratensis* AGMB00912 contained no antibiotic resistance genes. Additional HGT analysis showed that this genome had total five predicted transfers as a recipient. The five genes included two genes encoding 50S ribosomal protein (AGMB00912_00873 and 001346), one gene for 30S ribosomal protein synthesis (AGMB00912_00874), *ftsA* for cell division protein FtsA (AGMB00912_01862), and one hypothetical protein (AGMB00912_01967). These genes were predicted to be non-virulent and non-resistant, suggesting that AGMB00912 is safe in terms of HGT. The results of the virulence factor database, CARD, and HGT analyses expected the safety of AGMB00912 as a commercial resource. To validate its expectation, *in vitro* assays for antibiotic resistance, cytotoxicity, and hemolysis were conducted. In antibiotics resistance analysis, AGMB00912 was susceptible to ampicillin, gentamycin, kanamycin, streptomycin, erythromycin, tetracycline, and clindamycin, however exhibited resistance to chloramphenicol (Supplementary Table S6). Considering there was not resistance genes for chloramphenicol in CARD results, this resistance might be due to potential genetic mutation(s), suggesting its potential use despite the observed resistance. In cytotoxicity assay, AGMB00912 was non-cytotoxic compared to *E. coli* O157:H7 ATCC 48395, a known cytotoxic pathogen (Supplementary Figure S4A). In addition, AGMB00912 showed alpha-hemolysis (Supplementary Figures S4B–E). Previous review indicated that some probiotics demonstrate alpha-hemolytic activities (Goldstein et al., 2015), suggesting AGMB00912 could be considered safe for use.

### 3.4 LA supplementation promotes growth performance and protects against ETEC K88ac infection in weaning mice

**Figure 6:**
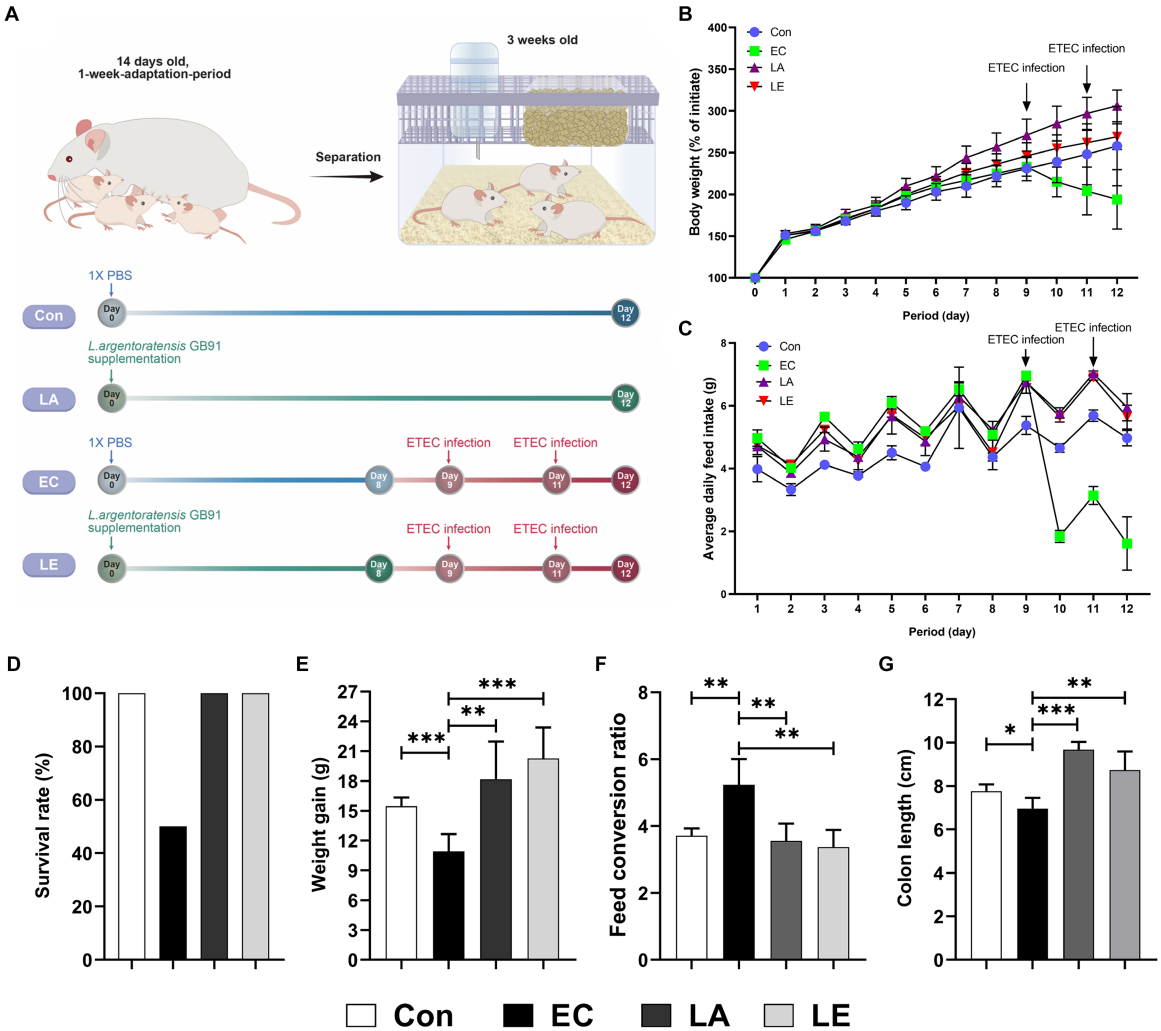
Effects of *L. argentoratensis* AGMB00912 on weaning mice: **(A)** Schematic diagram of the animal experiment. **(B)** Body weight change. **(C)** Average feed intake. **(D)** Survival rate. **(E)** Feed conversion ratio. **(F)** Weight gain. **(G)** Colon length. The data present the mean ± standard deviation (SD). Survival rate **(D)** was assessed using *n* = 10, while other measurements **(B,C,E,F,G)** were assessed using *n* = 5. Statistical significance was calculated using the Student’s two-tailed *t*-test (*p < 0.05, **p < 0.01, ***p < 0.001). Control, oral gavage with PBS; EC, enterotoxigenic *Escherichia coli* (10^9^ CFU/every 2 d); LA, *L. argentoratensis* AGMB00912 (10^9^ CFU/CFU/every 2 d); LE, LA plus EC (10^9^ CFU/every 2 d).

We investigated the protective effects of *Lactiplantibacillus argentoratensis* AGMB00912 (LA) on weaning mice infected with ETEC K88ac. The study included four groups: a control group, an ETEC-infected group (EC), an LA-supplemented group, and a group pretreated with LA before ETEC infection (LE) (Figure 6A). The EC group infected with ETEC K88ac displayed a significant decline in body weight, food intake, and weight gain compared with the control group, with a survival rate of 50% (Figure 6). However, the group supplemented with LA exhibited enhanced weight gain, feed intake, and feed conversion ratio compared with the control and EC groups. Additionally, the LE group infected with EC showed a significant increase in weight gain and food intake compared with the EC group; however, mortality was unaffected. Colon length was significantly reduced in the EC group relative to that in the control group (*p* < 0.05), whereas it was notably increased in the groups treated with LA (LA and LE; Figure 6G). These results suggest that LA treatment provides protection to ETEC-infected weaning mice, as demonstrated by the amelioration of growth performance and the absence of increased mortality.

### 3.5 LA supplementation regulates blood profile, serum cytokine, and antioxidant levels in ETEC-infected weaning mice

**Figure 7:**
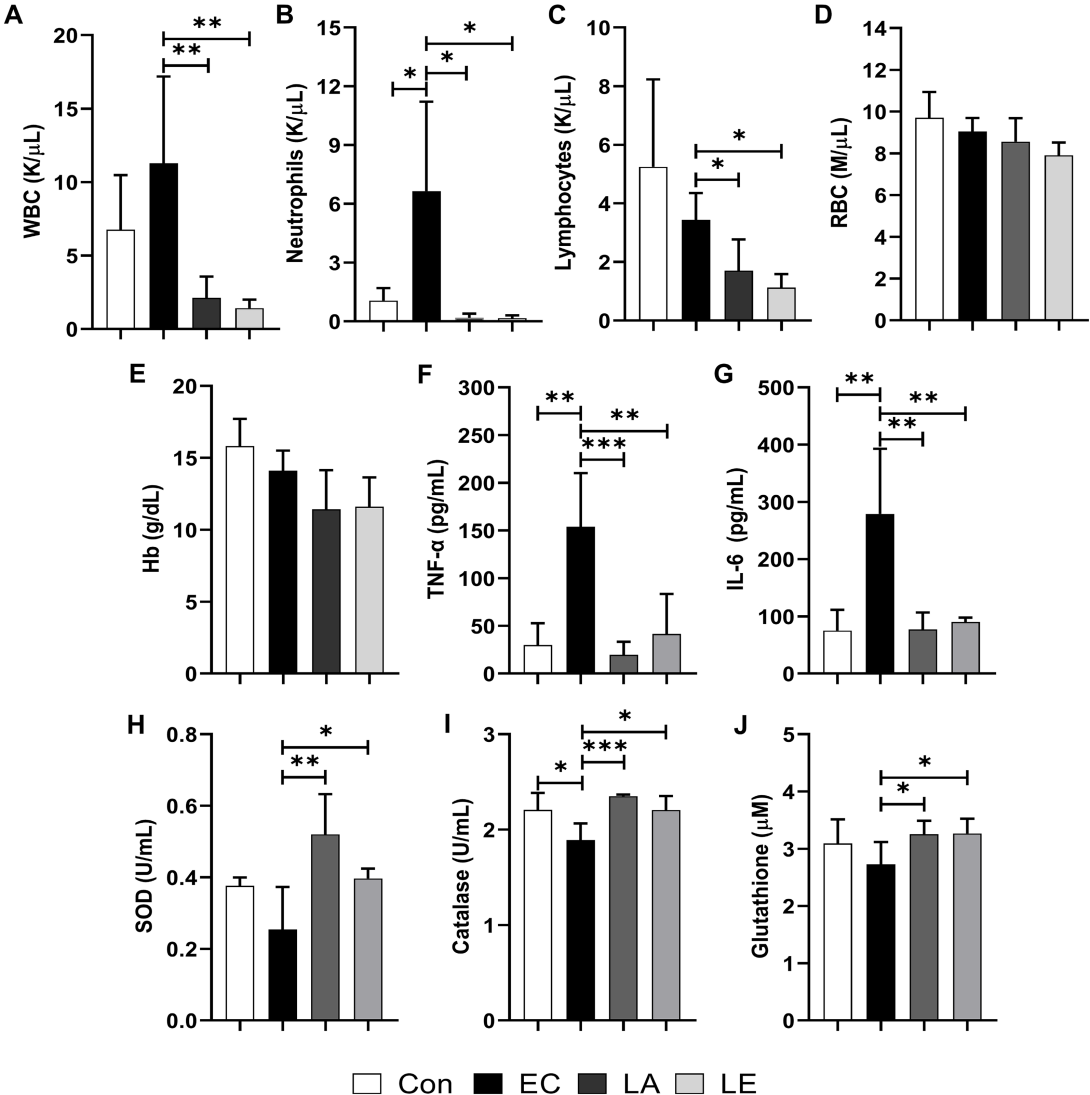
Effects of LA on blood biochemical, cytokine, and antioxidant index in weaning mice: **(A)** White blood cells. **(B)** neutrophils, **(C)** lymphocytes, **(D)** red blood cells, **(E)** hemoglobin, **(F)** TNF-α, **(G)** IL-6, **(H)** SOD, **(I)** catalase, and **(J)** glutathione levels. The data present mean ± SD (*n* = 5). Statistical significance was calculated using the Student’s two-tailed *t*-test (*p < 0.05, **p < 0.01, ***p < 0.001). Control, oral gavage with PBS; EC, enterotoxigenic *Escherichia coli* (10^9^ CFU/every 2 d); LA, *L. argentoratensis* AGMB00912 (10^9^ CFU/CFU/every 2 d); LE, LA plus EC (10^9^ CFU/every 2 d).

As shown in Figures 7A–C, the white blood cell, neutrophil, and lymphocyte counts in the LA supplementation groups (LA and LE) were significantly lower than in the ETEC treatment group. However, no significant differences in the levels of red blood cells or Hb were detected (Figures 7D,E). Compared to the control group, the levels of pro-inflammatory cytokines (TNF-α, IL-6) significantly increased in the ETEC-treated group (Figures 7F–G). Conversely, LA treatment downregulated their serum levels. Furthermore, antioxidant analysis revealed that weaning mice challenged with LA had significantly higher levels of superoxide dismutase, catalase, and glutathione (Figures 7H–J). These results indicate that supplementation with LA modulates the immune response, reducing the white blood cell, neutrophil, and lymphocyte counts, while also enhancing the antioxidant capacity of weaning mice, potentially providing a protective effect against ETEC-stimulated inflammation and oxidative stress.

### 3.6 LA supplementation promotes the SCFA production of weaning mice

**Figure 8:**
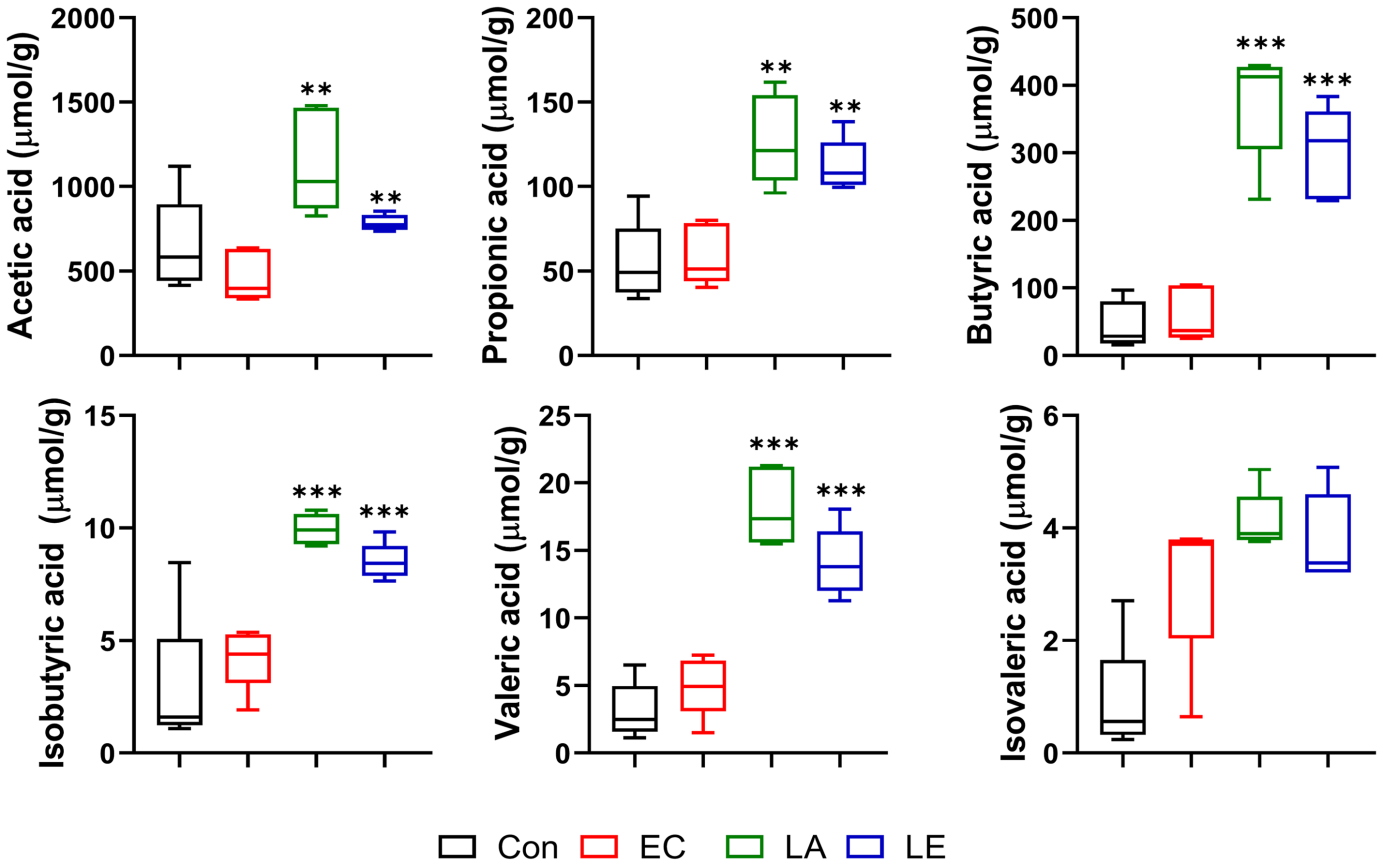
Short-chain fatty acid content of feces in different groups of mice. The data present the mean ± SD (*n* = 5). Statistical significance was calculated using the Student’s two-tailed *t*-test (*p < 0.05, **p < 0.01, ***p < 0.001). Control, oral gavage with PBS; EC, enterotoxigenic *Escherichia coli* (10^9^ CFU/every 2 d); LA, *L. argentoratensis* AGMB00912 (10^9^ CFU/CFU/every 2 d); LE, LA plus EC (10^9^ CFU/every 2 d).

The SCFAs produced by fermentation mediated by microorganisms in the intestines are crucial in nutrient digestion and absorption. We found that LA treatment promoted the production of acetic, propionic, butyric, iso-butyric, and valeric acids in fecal samples from the LA and LE groups (Figure 8). However, there were no significant differences in isovaleric acid levels between the EC and LA treatment groups. Hence, dietary supplementation with LA affected the production of SCFAs in feces.

### 3.7 LA supplementation alleviates intestinal damages in ETEC K88ac infected mice model

**Figure 9:**
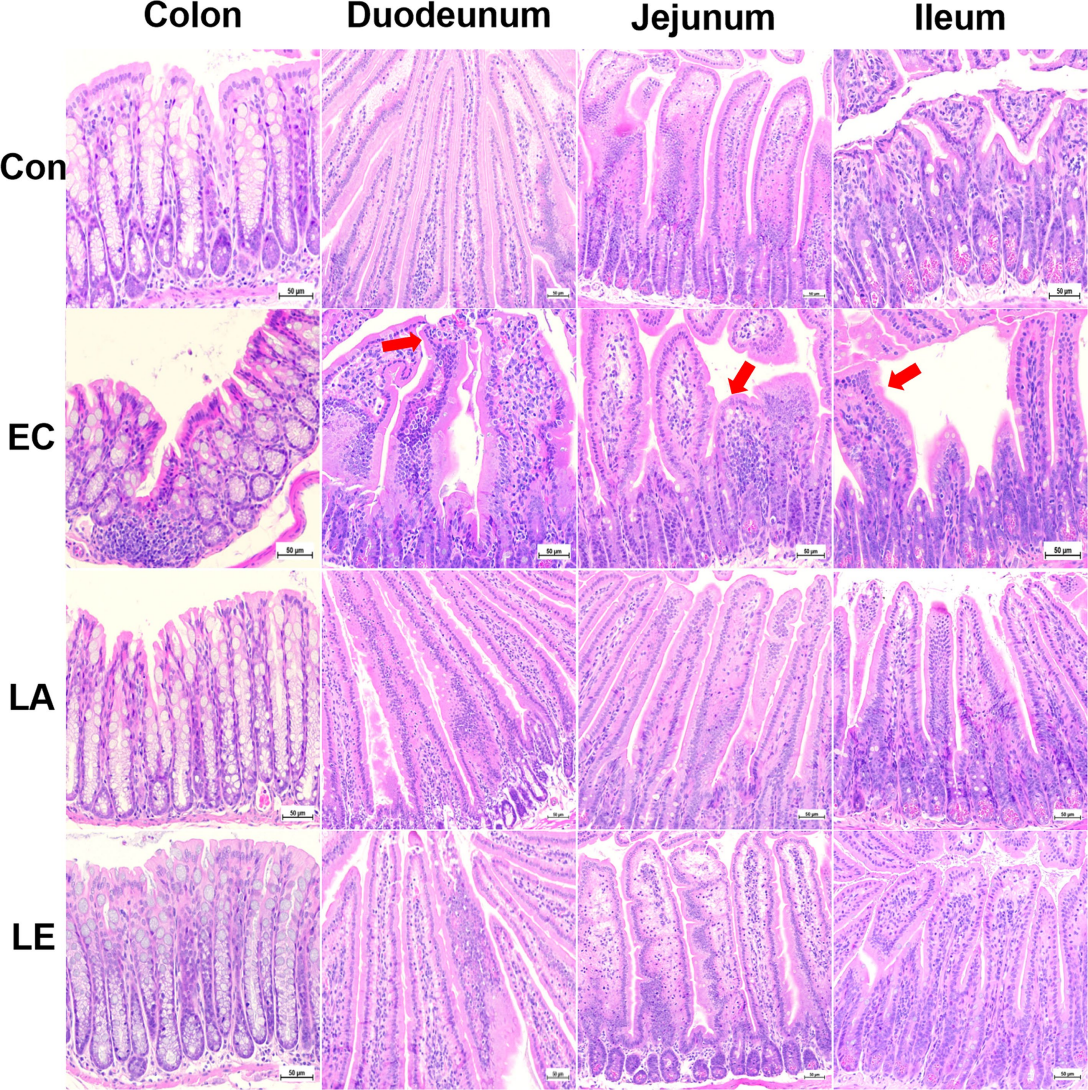
LA preserves intestinal morphology in ETEC-infected mice. Control, oral gavage with PBS; EC, enterotoxigenic *Escherichia coli* (10^9^ CFU/every 2 d); LA, *L. argentoratensis* AGMB00912 (10^9^ CFU/CFU/every 2 d); LE, LA plus EC (10^9^ CFU/every 2 d).

**Figure 10:**
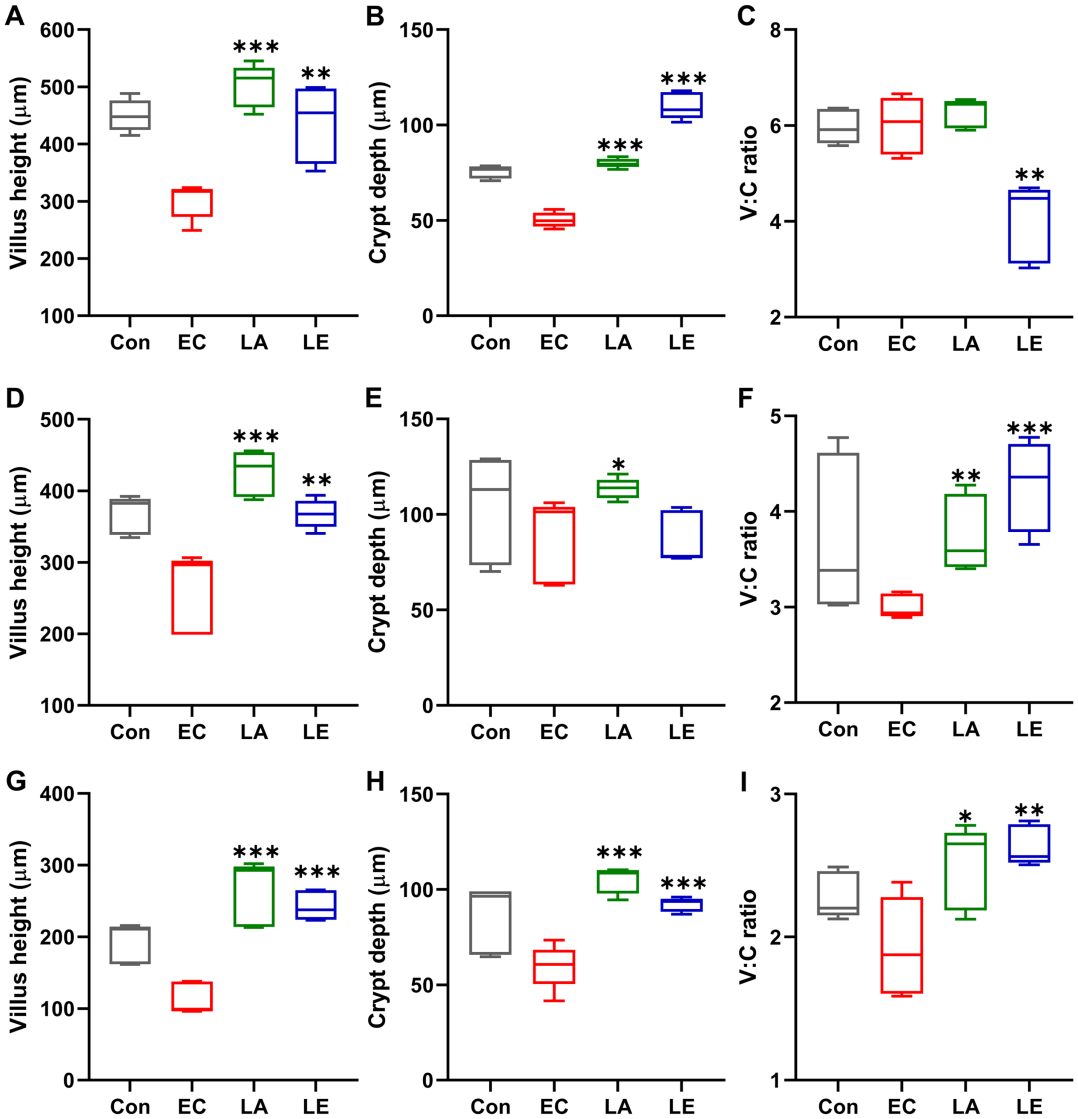
Villi length, crypt depth, and villi-to-crypt ratio in different groups of mice. The data present the mean ± SD (*n* = 5). Statistical significance was calculated using the Student’s two-tailed *t*-test (*p < 0.05, **p < 0.01, ***p < 0.001). Control, oral gavage with PBS; EC, enterotoxigenic *Escherichia coli* (10^9^ CFU/every 2 d); LA, *L. argentoratensis* AGMB00912 (10^9^ CFU/CFU/every 2 d); LE, LA plus EC (10^9^ CFU/every 2 d). **(A,D,G)** represent duodenum; **(B,E,H)** represent jejunum; and **(C,F,I)** represent ileum.

Figure 9 displays the analysis results of small and large intestinal morphology in ETEC-infected weaning mice. Microscopic analyses revealed that ETEC K88ac infection damaged the villi in the duodenum, jejunum, ileum, and colon tissues of mice in the EC groups, as evidenced by broken and shortened microvilli (indicated by red arrows). Additionally, there was an increase in inflammatory cell infiltration in these tissues. However, disruption of the villi and increased inflammatory cells in the small and large intestines were not observed in weaning mice in the LA group. Consistent with the results of microscopic analysis, ETEC K88-challenged groups significantly reduced villi height and crypt depth (Figure 10), and the V:C ratio in the duodenum, jejunum, and ileum tissues compared with the control group. Meanwhile, the LA groups (LA and LE) had significantly higher villi height and V:C ratio than weaning mice in the EC group. These results suggest that LA prevents intestinal destruction in weaning mice caused by ETEC infection.

### 3.8 LA supplementation modulates the gut microbiota composition of ETEC-infected weaning mice

**Figure 11:**
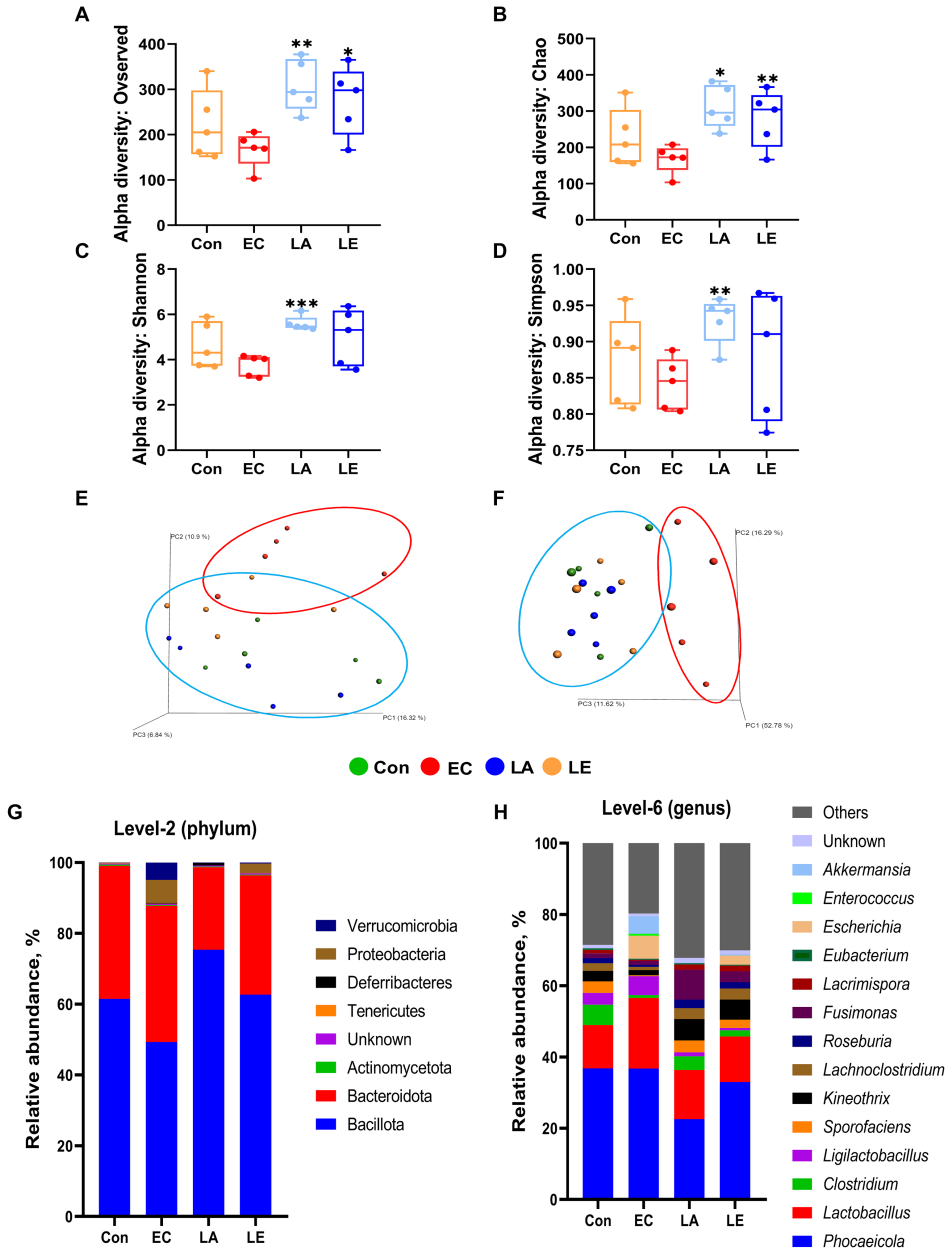
Box plots of the alpha diversity indices in mouse gut microbiotas treated with LA and EC. Species richness measured using **(A)** observed features and **(B)** Chao1 diversity indices. Species evenness measured using **(C)** Shannon and **(D)** Simpson diversity indices. The data present the mean ± SD (*n* = 5). Statistical significance for alpha diversity was calculated using the Student’s two-tailed t-test (*p < 0.05, **p < 0.01, ***p < 0.001). Principal coordinate analysis (PCoA) plots of different groups of mice. In week 2, EC group (red oval) and Con, LA, and LE group (blue oval) significantly clustered based on unweighted **(E)** and weighted **(F)** UniFrac distance metrics. Each group consisted of *n* = 5 replicates. Beta diversity significance was assessed using PERMANOVA, with p-values of 0.037 for unweighted and 0.745 for weighted UniFrac distances. Mouse gut microbiota composition at the phylum and genus level with LA and EC treatment. Bar plots show the relative abundance of each mouse group taxa at the phylum **(G)** and genus **(H)** levels.

To investigate the effects of LA on intestinal microbiota diversity in mice subjected to ETEC K88ac infection, we analyzed the V3–V4 region of the 16S rRNA gene amplicon sequencing in fecal samples. Sequencing of the 16S rRNA genes from the fecal samples yielded a range of reads across different groups. The control group had 138,730 to 165,900 reads per sample, the LA group had 147,322 to 191,784 reads per sample, the EC group had 131,996 to 161,780 reads per sample, and the LE group had 144,580 to 189,556 reads per sample (Supplementary Table S7). The alpha diversity indices (observed features, Chao1, Shannon, Simpson) in the fecal samples of weaning mice infected with ETEC K88ac are shown in Figures 11A–D. The LA and LE groups exhibited significantly higher alpha diversity, and the Chao 1 index was significantly higher than in the EC groups (*p* < 0.05) (Figures 11A,B). The LA groups showed a significant increase in the Shannon and Simpson indices compared with the ETEC-challenged group (*p* < 0.01); no significant difference was observed in the LE group (*p* > 0.05). Additionally, we performed community structure analysis (beta diversity) using PCoA based on unweighted (Figure 11E) and weighted UniFrac (Figure 11F) distances, showing significant differences in the separation of bacterial communities among the groups (EC, Con, LA, and LE) of weaning mice. Furthermore, we evaluated the grouping strength using permutational multivariate PERMANOVA. The PCoA plots derived from unweighted (Figure 11E) and weighted (Figure 11F) UniFrac distances revealed differences in the separation of microbial communities between the EC group and the other groups of weaning mice (*p* = 0.037 and 0.745, respectively). These results indicate that while ETEC K88 infection reduces the diversity of the gut microbiota, dietary supplementation with LA alleviates gut dysbiosis, with significant differences observed in the bacterial communities of weaning mice between the LA treatment groups (LA and LE) and the EC group.

We examined the relative abundance of the microbial community in weaning mice treated with LA or ETEC (Figures 11G–H). At the phylum level, the EC group showed a decrease in Bacillota from 61.49 to 49.31% compared to the control group, whereas the proportions of Proteobacteria and Verrucomicrobiota increased from 0.03 to 6.53% and from 0.00 to 4.89%, respectively. The LA and LE groups exhibited Bacillota proportions of 75.38 and 62.69%, and Proteobacteria and Verrucomicrobiota had proportions of 0.01, 2.62, 0.06, and 0.28%, respectively (Figure 11G). At the genus level, *Phocaeicola*, *Lactobacillus*, *Clostridium*, *Ligilactobacillus*, *Sporofaciens*, *Kineothrix*, *Lachnoclostridium*, *Roseburia*, *Fusimonas*, *Lacrimispora*, *Eubacterium*, *Escherichia*, *Enterococcus*, and *Akkermansia* accounted for >70% of all microbial communities. Notably, the relative abundance of the genera *Escherichia* and *Akkermansia* also increased in the EC groups compared with the control group, whereas they decreased with LA supplementation, a similar pattern to that observed for Proteobacteria and Verrucomicrobiota. This finding suggests that *Escherichia* and *Akkermansia* may be the main genera contributing to the changes in Proteobacteria and Verrucomicrobiota (Figure 11H).

**Figure 12:**
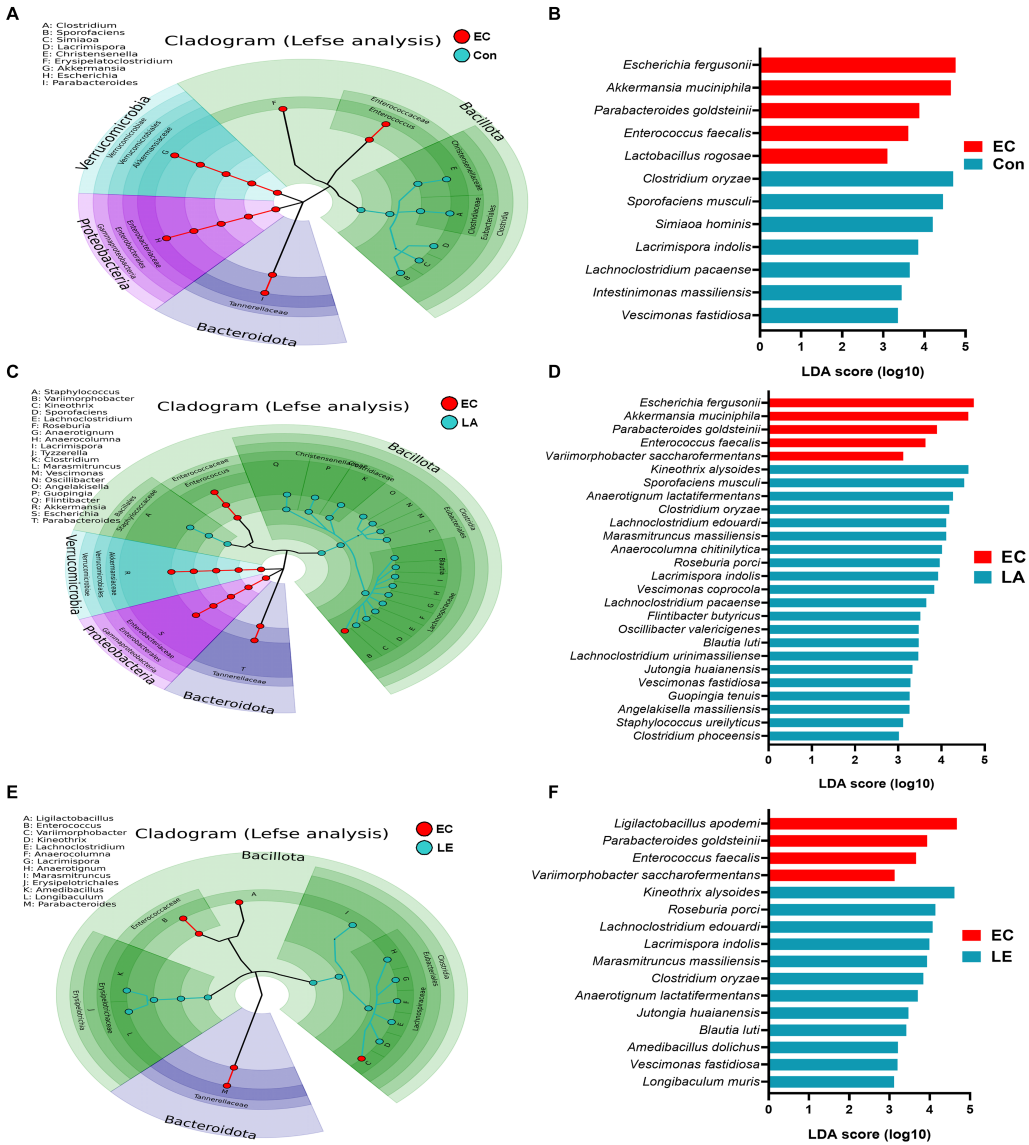
Differential abundance of bacteria among the EC group determined using the linear discriminant analysis effect size (LEfSe) algorithm. A *p* < 0.05 was deemed significant in the Kruskal–Wallis and Wilcoxon tests. The cladogram shows differential abundance at the phylum, class, order, family, and genus levels **(A–C)**. Histogram of differential abundance at the species level **(D–F)**. A discriminative feature had a log_10_ linear discriminant analysis (LDA) score of 3. The length of each histogram represents the LDA score, indicating the degree of influence for each species.

**Figure 13:**
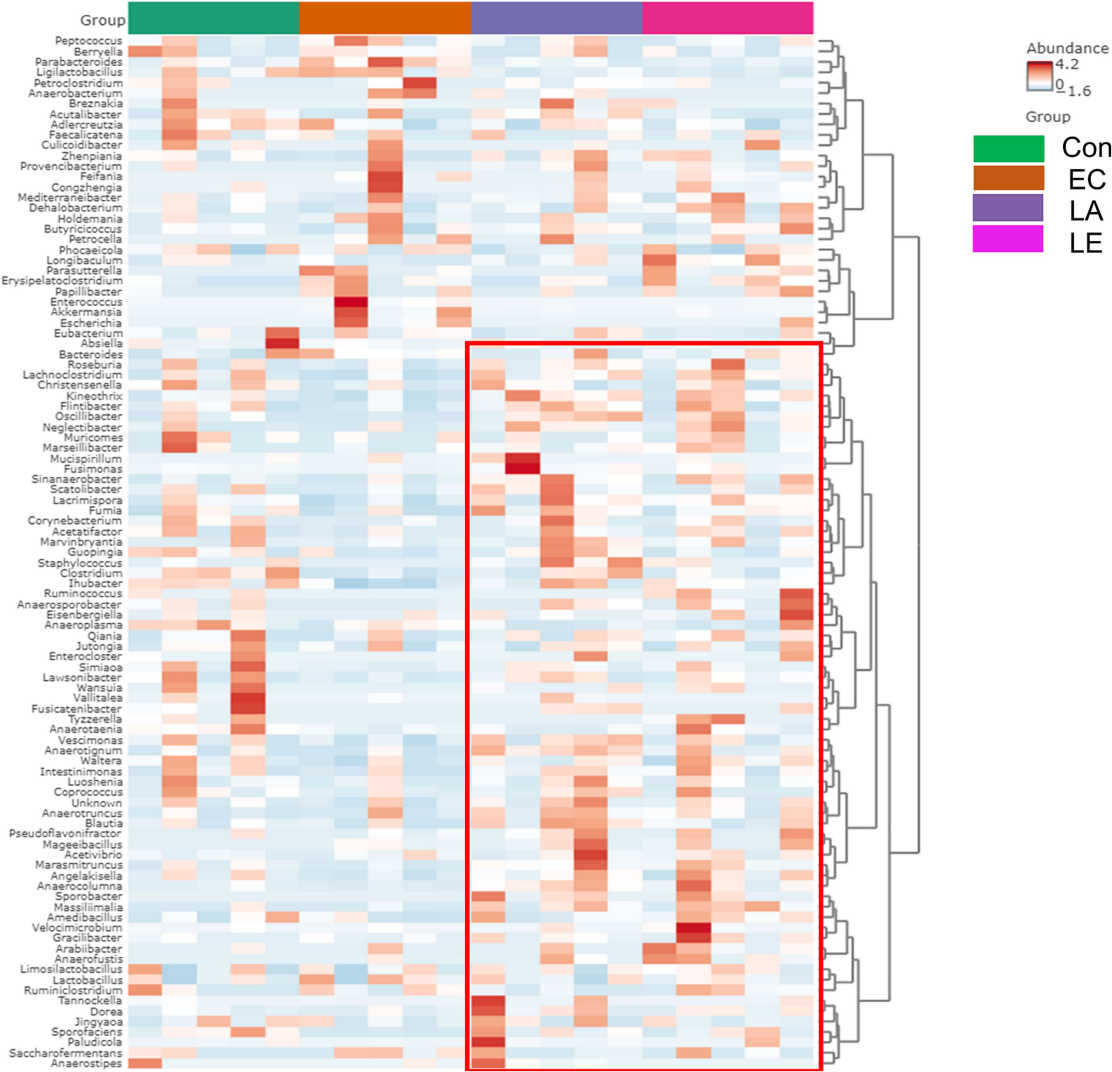
Hierarchical clustering heatmap at the genus level using Ward and Euclidean parameters. Red represents high abundance, and blue represents low abundance. The vertical axis represents the taxonomy level, and the horizontal axis represents the aggregated individuals according to the treatment. The red rectangle represents the unique cluster with higher relative taxa abundance in the EC group compared with the LA and LE groups.

To verify the significantly different taxa of gut microbiota among the various groups (control vs. EC, EC vs. LA, and EC vs. LE), the LEfSe algorithm was used (Figure 12). The LEfSe analysis-based cladogram was visualized for the phylum to genus levels (Figures 12A–C), and the histogram (Figures 12D–F) exhibited a significantly different abundance at the species level, as indicated by the LDA score (LDA > 3). The LEfSe results indicated that the abundance of *Escherichia*, *Akkermansia*, *Parabacteroides*, and *Enterococcus* was significantly higher in the EC group compared to the control group (*p* < 0.05), as shown in Figures 12A,D. This trend was consistent with the relative abundance data presented in Figures 11G,H. Conversely, LA supplementation (LA and LE) significantly increased the relative abundance of *Kineothrix*, *Lachnoclostridium*, *Roseuburia*, *Lacrimispora*, *Jutongia*, *Marasmitruncus*, *Clostridium*, *Blautia*, and *Vescimonas*, while decreasing the abundance of *Escherichia* and *Enterococcus* (*p* < 0.05; Figures 12B–F). To enhance the visualization of the varying abundances of bacterial genera across the treatment groups, a hierarchical clustering heat map was generated (Figure 13).

Analysis of the core microbiome was conducted at the genus level, utilizing sample prevalence and relative abundance thresholds of 20 and 0.02%, respectively. In all experimental groups, four core bacterial genera were identified: *Phocaeicola*, *Lactobacillus*, *Limosilactobacillus*, and *Kineothrix*. However, *Escherichia*, *Akkermansia*, and *Parabacteroides* were found exclusively in the EC group (Supplementary Figure S5). These results indicated that dietary supplementation with LA significantly modulated the gut microbiota composition in weaning mice challenged with ETEC K88ac infection.

### 3.9 LA supplementation regulates the gut-related metabolic function in weaning mice

**Figure 14:**
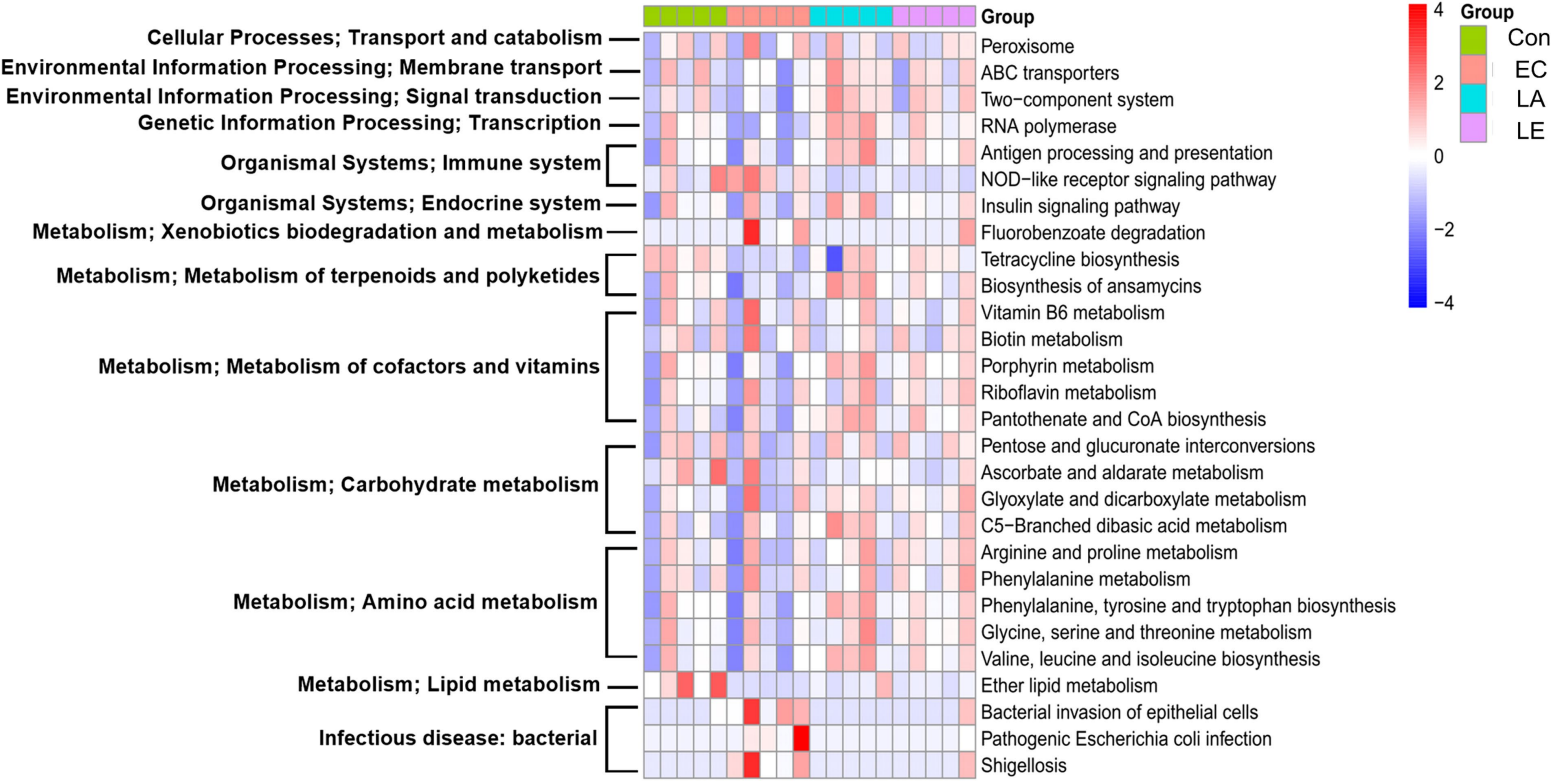
Effect of LA on gut microbiota that regulate functional pathways in ETEC K88ac infected mice. PICRUSt2 software functional pathway prediction based on the fecal 16S ribosomal RNA V3–V4 gene expression analysis. Heat map of different functional pathways between the four groups. Differences in the predicted metagenomic functions between the groups were analyzed using the R software package ALDEx2, with significance set at *p* < 0.05.

To predict the metabolic function of the differential gut microbiota in weaning mice treated with LA, a functional assessment using PICRUSt2 was performed to analyze KEGG pathway abundance. The heatmap identified pathways in the top 12 categories, including transport and catabolism, membrane transport, signal transduction, immune system, endocrine system, infectious disease, xenobiotics, terpenoids, cofactors, vitamins, carbohydrates, amino acids, and lipid metabolism, affected by the gut microbiota in each group (Figure 14). Supplementary Figure 6 shows 28 subcategories of differential metabolism. Compared to the control group, metabolic pathways associated with the NOD-like receptor signaling pathway, bacterial invasion of epithelial cells, shigellosis, and pathogenic *E. coli* infection were found to be upregulated in the group infected with ETEC. In contrast, LA treatment significantly attenuated the ETEC-induced upregulation of these pathways (*p* < 0.05). Additionally, LA treatment upregulated the metabolism of several pathways, including ansamycins, cofactors, and vitamins (vitamin B6, biotin, porphyrin, riboflavin, pantothenate, and CoA), carbohydrates (glyoxylate, dicarboxylate, and C5-branched dibasic acid), and amino acids (arginine, proline, phenylalanine, tyrosine, tryptophan, glycine, serine, threonine, valine, leucine, and isoleucine) biosynthesis. These results indicate that LA treatment significantly attenuated the ETEC-induced upregulation of detrimental metabolic pathways while enhancing the metabolism of ansamycins, vitamins, carbohydrates, and amino acids, suggesting a potential protective and restorative effect on gut microbiota metabolism during ETEC infection.

## 4 Discussion

In this study, we isolated the LA strain from healthy swine. The CFS of LA demonstrated antimicrobial activity against ETEC, STEC, and ST454 in well-diffusion and 96-well-broth assays. Additionally, LA inhibited the growth of seven pathogens in a co-culture assay, primarily due to its metabolites. Probiotics exhibit antagonistic properties against pathogens by producing an acidic environment via SCFA synthesis and secreting reactive oxygen species such as hydrogen peroxide and other antibacterial compounds (Delley et al., 2015; Benavides et al., 2016; Wang T. et al., 2018; Hassan et al., 2020). Although the antimicrobial activity of LA CFS was not affected by heat or enzyme treatment, it was attenuated by pH adjustment. This suggests that the antagonistic activity of LA is due to the acidic environment created by the SCFAs, consistent with findings by Hassan et al. (2020) and Mlambo et al. (2022).

Growth performance during the weaning period is crucial in livestock production, and infections by pathogens like ETEC can severely impact this process by reducing body weight and feed intake, while also increasing mortality rates (Gresse et al., 2017; Tao et al., 2018; Cho and Yoon, 2014; Pereira et al., 2022). Specifically, ETEC infection leads to intestinal injury, characterized by shortened villi height, expanded crypt depth, and a decreased V:C ratio, all of which hinder nutrient absorption (Ren et al., 2014; Wei et al., 2023). The small intestine is crucial for nutrient absorption and growth during weaning, with villi expanding the contact area between the intestine and nutrients, promoting absorption and physically separating the organ from pathogens and harmful compounds (Caspary, 1992; Yun et al., 2020; Rose et al., 2021). Our study aimed to evaluate the protective effects of LA, particularly focusing on its antibacterial and SCFA-producing activities, using an ETEC K88ac-infected weaning mouse model. The results confirmed that ETEC K88ac infection significantly compromised growth performance in weaning mice, as evidenced by decreased body weight gain, reduced feed intake, and increased feed conversion ratio and mortality. These detrimental effects can be attributed to the enterotoxins produced by ETEC, which disrupt intestinal integrity, impair fluid and nutrient absorption, and induce apoptosis (Yang et al., 2016; Moeser and Blikslager, 2007; Ghosal et al., 2013; Johnson et al., 2010; Nakashima et al., 2013; Syed and Dubreuil, 2012). In contrast, dietary supplementation with LA demonstrated a protective effect by significantly enhancing body weight gain and feed intake, reducing the feed conversion ratio, and preventing mortality due to ETEC infection. These findings are consistent with other studies showing that probiotic treatments can alleviate ETEC infection symptoms and improve growth performance (Wang T. et al., 2018; Liu et al., 2017). Furthermore, LA supplementation prevented colon shortening—a hallmark of ETEC infection (Wang et al., 2022)—and preserved the microvilli of the small and large intestines, resulting in improved villi height and V:C ratio in weaning mice. These protective effects are further supported by Wei et al. (2023), who reported that probiotics enhance intestinal barrier integrity and strengthen the epithelial lining.

ETEC infection typically causes a rapid increase in leukocytes, neutrophils, and lymphocytes (Dubreuil et al., 2016). ETEC infection promotes pro-inflammatory cytokine activation via neutrophil transmigration (Roselli et al., 2006). This pro-inflammatory cytokine production suppresses cellular redox status, further damaging intestinal function (Vergauwen et al., 2015; Rosero et al., 2015). However, LA supplementation significantly decreased white blood cell, neutrophil, and lymphocyte counts and pro-inflammatory cytokine levels (TNF-α, interleukin-6) in ETEC-challenged mice. Moreover, intestinal inflammation is associated with oxidative stress. Thus, enhancing antioxidant activity is crucial for healthy livestock growth and development. The acetate-producing pathway utilized by LA forms carbon dioxide and hydrogen peroxide, potentially improving antioxidant activity (Christensen et al., 1999). Indeed, LA significantly increased superoxide dismutase, catalase, and glutathione levels, suggesting boosted antioxidant activity and reduced intestinal inflammation. Similarly, *L. rhamnosus* and *L. acidophilus* treatments have also been shown to improve antioxidant capacity in *Salmonella*-challenged rats (Moorthy et al., 2007).

Previous studies have shown that *Lactiplantibacillus* spp. administration to mice increases SCFAs in the gut, altering pH and the intestinal microbiome (Hong et al., 2021; Bu et al., 2023; Scott et al., 2008; Oh et al., 2021). Genetic analysis confirmed that LA produces SCFAs, particularly lactate, acetate, and formate. The levels of acetic, propionic, butyric, iso-butyric, and valeric acids in fecal samples from the LA and LE groups were significantly increased compared to the EC group. Chen et al. (2023) also reported that *L. gasseri* supplementation increased SCFA levels in fecal samples and alleviated clinical symptoms in ETEC-infected mice. SCFAs, including acetate, propionate, and butyrate, along with carboxylic acids with aliphatic tails shorter than six carbon atoms, are primarily produced through anaerobic microbial fermentation of dietary fibers, resistant starch, and proteins in the cecum and colon (Koh et al., 2016; Granado-Serrano et al., 2019). Acetate can be enzymatically converted to butyrate via butyryl-CoA: acetyl-CoA transferase (Duncan et al., 2002). Meanwhile, acetate protects against *E. coli* O157 infection, modulates intestinal permeability in mice (Fukuda et al., 2011), and mitigates lipopolysaccharide-induced diarrhea in mice by regulating tight junction protein expression in porcine intestinal epithelial cells (Andrani et al., 2023). Propionate and butyrate provide energy to intestinal epithelial cells, activate PPAR-γ signaling (Wang et al., 2015), stimulate epithelial cells, and promote colonocyte metabolism through coordination with Tregs (Byndloss and Bäumler, 2018; den Besten et al., 2015). Our study suggests that the SCFA-producing ability of LA correlates with increased SCFA content in mouse feces and alleviates pathogenic symptoms.

Mammalian intestinal microbiota play a vital role in host health by maintaining gut homeostasis, modulating immune responses, fermenting and absorbing nutrients, and providing protection against pathogens (Buffie and Pamer, 2013; Kamada et al., 2013; Fujiwara et al., 2018; Menghini et al., 2019). However, the stability and diversity of intestinal microbiota can be disrupted by pathogenic microorganisms, leading to weakened intestinal barrier integrity, upregulation of inflammatory responses, and gut dysbiosis (Li et al., 2021; Chen et al., 2022). Our findings indicate that ETEC K88 infection decreased the alpha diversity of microbes in weaning mice, causing an imbalance in the intestinal microbiota by colonizing the small intestine and secreting enterotoxins (Kataoka, 2016). Dietary supplementation with LA mitigated this disruption of microbial diversity. Previous studies have similarly reported that probiotic bacteria can reduce the disruption of bacterial community diversity and richness caused by ETEC infection (Yue et al., 2020; Li et al., 2022). ETEC infection also significantly increased the relative abundance of harmful bacteria such as Proteobacteria and Verrucomicrobiota while reducing beneficial bacteria like Bacillota. An increase in Proteobacteria, including *Escherichia* and *Salmonella*, is a typical indicator of intestinal disorders caused by ETEC infection (Yang et al., 2016; Rizzatti et al., 2017; Litvak et al., 2017). *E. coli* challenges can also increase the relative abundance of *Escherichia*, *Akkermansia*, and *Enterococcus* spp. in mice (Chen et al., 2023; Bian et al., 2016; Wei et al., 2020). Nitric oxide produced during intestinal inflammation is converted into nitrate, promoting the growth of *Escherichia* spp. due to its nitrate reductase gene (Spees et al., 2013; Bäumler and Sperandio, 2016). Additionally, increased blood flow in the inflamed intestine leads to oxygen accumulation, favoring facultative anaerobes like Proteobacteria, disrupting anaerobic conditions, and reducing bacterial diversity (Yang et al., 2016; Zeng et al., 2017). Meanwhile, LA dietary supplementation reduced the abundance of Proteobacteria and increased Bacillota, crucial for gut health (Sun et al., 2019; Elokil et al., 2020). Moreover, LA supplementation increased the proportion of SCFA-producing bacteria, including *Kineothrix*, *Lachnoclostridium*, *Roseburia*, *Lacrimispora*, *Jutongia*, and *Blautia*. *Kineothrix* produces saccharolytic butyrate, which has beneficial therapeutic effects (Canani et al., 2011; Haas and Blanchard, 2017; Liddicoat et al., 2020). *Lachnoclostridium* produces butyrate via the 4-aminobutyrate/succinate pathway from acetate and lactate (Van den Abbeele et al., 2013; Vital et al., 2014) and has anti-inflammatory properties (Zhou et al., 2020). *Roseburia* regulates glycolipid metabolism and butyric acid production, alleviating inflammatory bowel diseases and promoting immune system maturation (Jost et al., 2013; Takahashi et al., 2016; La Rosa et al., 2019). *Lacrimispora*, reclassified under Lachnospiraceae, produces butyrate via the acetyl-CoA pathway (Vital et al., 2014; Haas and Blanchard, 2020), and its abundance correlates positively with acetate and butyrate levels in feces (Holst et al., 2022). *Jutongia* synthesizes large amounts of butyric acid, promoting host immunity and modulating inflammation (Wei et al., 2020). *Blautia*, an anaerobic bacterium, alleviates inflammatory diseases, prevents gut inflammation, and maintains the intestinal microbial community by producing SCFAs (Kim et al., 2014; Khattab et al., 2017; Kalyana Chakravarthy et al., 2018; Liu et al., 2021). Additionally, it can decrease cecal pH and increase SCFA levels in rats (Minamida et al., 2005). Our findings suggest that LA supplementation alleviates the negative effects of ETEC-K88 infection on the gut microbiota of weaning mice by reducing harmful microorganisms and increasing beneficial SCFA-producing bacteria. This microbial shift enhances host immunity and modulates inflammatory responses against ETEC infection.

To predict the metabolic function of the differentially abundant gut microbiota, we analyzed the KEGG gene function using PICRUSt2 based on 16S rDNA gene sequences. LA supplementation significantly increased the abundance of carbohydrate, amino acid and cofactors and vitamins metabolism compared to the EC group. Carbohydrate metabolism is crucial for digestion, absorption, and fermentation by the gut microbiota to produce SCFAs. Liu et al. (2020) suggested that glyoxylate and dicarboxylate metabolism is positively correlated with acetic acid secretion, and C5-branched dibasic acid metabolism is essential for supplying energy for cellular metabolic activities (Yuan et al., 2020). Amino acids such as valine, leucine, isoleucine, phenylalanine, tyrosine, and arginine can be potential precursors of SCFAs and sources for the tricarboxylic acid cycle (Pratelli and Pilot, 2014; Neis et al., 2015; Neinast et al., 2019). Phenylalanine, tyrosine, tryptophan, and phenylalanine metabolism are linked to an increased abundance of beneficial bacteria and help prevent gut inflammation (Liu et al., 2018; Cong et al., 2023). Moreover, vitamin B6, riboflavin, biotin, pantothenate, and CoA are essential for cellular metabolism, with vitamin B6 contributing to fatty acid biosynthesis (Seki, 2006; Theodoulou et al., 2014; Kelekar and Jameson, 2021; Parra et al., 2018). The upregulation of the ABC transport system in the LA group, which aids in the conversion of sugars to SCFAs, helps inhibit enterotoxins and protect against enteropathogenic infection (Fukuda et al., 2011; Jeckelmann and Erni, 2020). These findings suggest that the predicted metabolic functions positively affect SCFA production.

LA treatment also significantly enhanced the biosynthesis of ansamycins, antibiotics with antimicrobial activity against bacteria, fungi, and viruses (Alghaithy et al., 2000). Bacterial invasion of epithelial cells, pathogenic *E. coli* infection, shigellosis, and NOD-like receptor signaling pathways were significantly downregulated in the LA group. Similarly, Sun et al. (2020) reported that the *L. gasseri* JM1 strain exerts immunomodulatory effects via the NOD2-mediated signaling pathway. NOD-like receptors recognize ligands in pathogenic microorganisms, activating inflammatory and immune responses (Motta et al., 2015).

**Figure 15:**
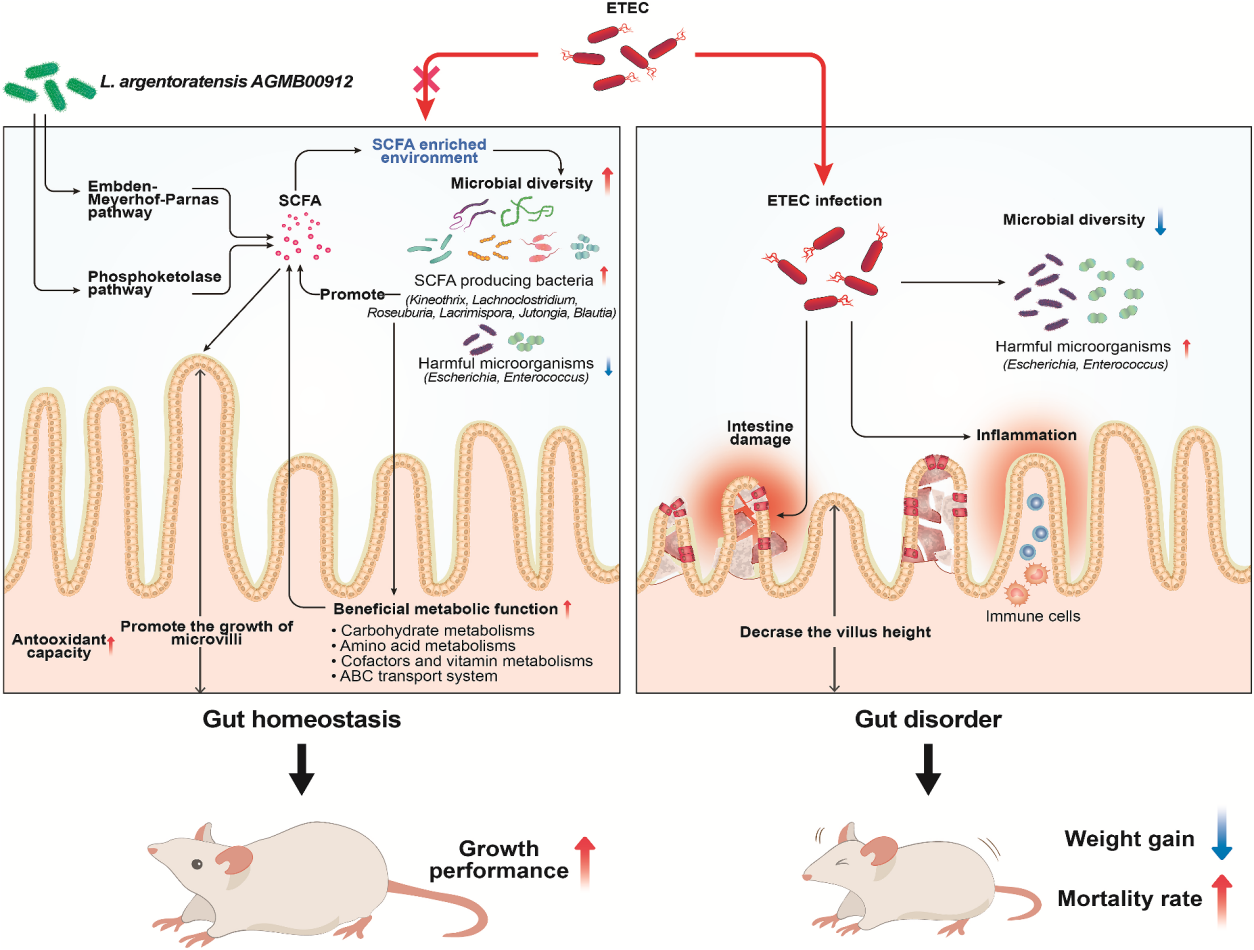
Schematic diagram illustrating the protective mechanisms of *Lactiplantibacillus argentoratensis* AGMB00912 in weaning mice challenged with enterotoxigenic *Escherichia coli* (ETEC). The pathways through which *L. argentoratensis* AGMB00912 enhances intestinal integrity, promotes SCFA production, and modulates the gut microbiota to confer resistance against ETEC infection are shown.

Based on our study, the microbial community analysis did not show a significant increase in the abundance of the Lactobacillus genus in the LA-supplemented groups. However, LA supplementation did increase the relative abundance of beneficial gut bacteria. These results demonstrate the potential influence of LA on the intestinal microbiota of weaning mice, particularly when challenged with ETEC K88. LA supplementation not only ameliorates the clinical symptoms induced by ETEC K88ac infection but also promotes the proliferation of SCFA-producing bacteria such as *Kineothrix* and *Lachnoclostridium*, enriching the gut environment with vital SCFAs that help mitigate inflammatory responses, enhance gut barrier integrity, and reduce the effects of pathogenic infections (Figure 15). The value of this new strain for pigs lies in its ability to enhance gut health through the production of SCFAs and other beneficial metabolites. Unlike other reported strains, LA has a unique ability to significantly modulate the gut microbiota and improve gut barrier functions. This is particularly valuable for the swine industry, where post-weaning diarrhea caused by pathogenic bacteria like ETEC can lead to significant economic losses.

The major limitation of the current study is the use of weaning mice instead of pigs. However, this model was selected for initial experiments due to its shorter gestation periods and lower maintenance costs, allowing for more rapid and controlled studies. Nevertheless, the ultimate goal is to apply these findings to pigs. Therefore, comprehensive evaluations within the swine industry are required to further elucidate these findings and explore the alleviation of gut health issues in weaning piglets.

## Data Availability

The datasets presented in this study can be found in online repositories. The names of the repository/repositories and accession number(s) can be found at: https://www.ncbi.nlm.nih.gov/genbank/, CP136431; https://www.ncbi.nlm.nih.gov/, PRJNA1040026.

## Glossary

AGMB: isolated gut bacteria
ANI: average nucleotide identity
BLAST: basic local alignment search tool
CARD: comprehensive antibiotic resistance database
CFS: cell-free supernatant
EMP: Embden–Meyerhoff–Parnas
ELISA: enzyme-linked immunosorbent assay
EFSA: European Food Safety Authority’s
ETEC: enterotoxigenic *E. coli*
HGT: horizontal gene transfer
IL: interleukin
KAAS: KEGG automatic annotation server
KEGG: Kyoto Encyclopedia for Genes and Genomes
KVCC: Korea Veterinary Culture Collection
LA: *Lactiplantibacillus argentoratensis* AGMB00912
LB: Luria–Bertani
LDA: linear discriminant analysis
LEfSe: linear discriminant analysis effect size
MRS: De Man-Rogosa-Sharpe
NCBI: National Center for Biotechnology Information
NOD: nucleotide oligomerization domain
NGS: next generation sequencing
ONT: Oxford Nanopore Technologies
PBS: phosphate-buffered serum
PCoA: principal coordinate analysis
PCR: polymerase chain reaction
PERMANOVA: permutational multivariate analysis of variance
SCFA: short-chain fatty acid
STEC: Shiga toxin-producing *E. coli*
ST454: *Salmonella Typhimurium*
TNF-α: tumor necrosis factor-α
