# Supplementary material for: Lactiplantibacillus argentoratensis AGMB00912 protects weaning mice from ETEC infection and enhances gut health
Source: Front Microbiol. 2024 Sep 10;15:1440134. doi: 10.3389/fmicb.2024.1440134 (PMC11420142; doi:10.3389/fmicb.2024.1440134)

Supplementary Material

**Supplementary Table 1.** Information on *Lactiplantibacillus* species used in the Average Nucleotide Identity analysis

| **Species** | **Strain** | **Accession number** | **GenBank Assembly** |
| --- | --- | --- | --- |
| *Lactobacillus pentosus* | BGM 48 | GCA_002850015.1 | ASM285001v1 |
|  | DSM 20314 | GCA_003641185.1 | ASM364118v1 |
|  | KW 1 | GCA_023972895.1 | ASM2397289v1 |
|  | ZFM 222 | GCA_003627295.1 | ASM362729v1 |
|  | 9D3 | GCA_023823145.1 | ASM2382314v1 |
| *Lactiplantibacillus paraplantarum* | L-ZS9 | GCA_001443645.1 | ASM144364v1 |
|  | FL-8 | GCA_029025825.1 | ASM2902582v1 |
|  | DSM 10667 | GCA_003641145.1 | ASM364114v1 |
|  | CK 401 | GCA_013113675.1 | ASM1311367v1 |
|  | KCCM 11826P | GCA_021869545.1 | ASM2186954v1 |
| *L. plantarum* subsp. *plantarum* | Lp 835 | GCA_009889735.1 | ASM988973v1 |
|  | Lp Ras | GCA_009889775.1 | ASM988977v1 |
|  | TMW 1.460 | GCA_009864015.1 | ASM986401v1 |
|  | FUA 3584 | GCA_009863935.1 | ASM986393v1 |
|  | SRCM 100442 | GCA_009913655.1 | ASM991365v1 |
| *L. plantarum* subsp. *argentoratensis* | AGMB00912 | CP136431 |  |
|  | XSBN-2 | GCA_017963485.1 | ASM1796348v1 |
|  | LQC 2422 | GCA_018551525.1 | ASM1855152v1 |
|  | DSM 16365 | GCA_001435215.1 | ASM143521v1 |
|  | NBRC 106486 | GCA_007991855.1 | ASM799185v1 |

**Supplementary Table 2.** Genomic information of *L. argentoratensis* AGMB00912 and *L. argentoratensis* strains used in the pan-genome analysis

| **GenBank Assembly** | **Accession number** | **Name** | **Size (bp)** | **GC content (%)** | **Total genes** | **CDS** | **rRNA** | **tRNA** |
| --- | --- | --- | --- | --- | --- | --- | --- | --- |
|  | CP136431 | AGMB00912 | 2,988,800 | 45.43 | 2,949 | 2,869 | 16 | 64 |
| ASM143521v1 | GCA_001435215.1 | DSM 16365^T^ | 3,350,338 | 45.33 | 3,285 | 3,184 | 16 | 84 |
| ASM1855230v1 | GCA_018552305.1 | LQC 2516 | 3,148,153 | 35.74 | 3,012 | 2,956 | 2 | 53 |
| ASM1855233v1 | GCA_018552335.1 | LQC 2485 | 3,494,755 | 41.74 | 3,347 | 3,284 | 5 | 57 |
| ASM799185v1 | GCA_007991855.1 | NBRC 106468 | 3,128,971 | 46.24 | 2,952 | 2,907 | 2 | 42 |
| ASM1796348v1 | GCA_017963485.1 | XSBN-2 | 3,119,902 | 46.32 | 3,017 | 2,955 | 2 | 59 |
| ASM1855156v1 | GCA_018551565.1 | LQC 2520 | 3,175,498 | 64.31 | 3,034 | 2,978 | 2 | 53 |
| ASM1855229v1 | GCA_018552295.1 | LQC 2320 | 3,181,752 | 45.20 | 3,036 | 2,980 | 2 | 53 |
| ASM1855159v1 | GCA_018551595.1 | LQC 2441 | 3,147,789 | 36.63 | 3,018 | 2,962 | 2 | 53 |
| ASM1855152v1 | GCA_018551525.1 | LQC 2422 | 3,128,861 | 35.22 | 2,999 | 2,943 | 2 | 53 |

**Supplementary Table 3.** Antimicrobial activity of 100 CFSs against pathogenic intestinal bacteria

| **Strains** | **Antimicrobial activity ^a^** | | | **Strains** | **Antimicrobial activity ^a^** | | | **Strains** | **Antimicrobial activity ^a^** | | |
| --- | --- | --- | --- | --- | --- | --- | --- | --- | --- | --- | --- |
|  | **ETEC** | **STEC** | **ST** |  | **ETEC** | **STEC** | **ST** |  | **ETEC** | **STEC** | **ST** |
| **AGMB00273** | - | - | - | **AGMB03517** | - | - | - | **AGMB00911** | - | - | - |
| **AGMB00119** | - | - | - | **AGMB02792** | - | - | - | **AGMB01810** | - | - | - |
| **AGMB00782** | - | - | - | **AGMB02776** | - | - | - | **AGMB01799** | - | - | - |
| **AGMB00347** | - | - | - | **AGMB03753** | - | - | - | **AGMB01710** | - | - | - |
| **AGMB00335** | - | - | - | **AGMB03644** | - | - | - | **AGMB00828** | - | - | - |
| **AGMB00339** | - | - | - | **AGMB03665** | - | - | - | **AGMB00184** | - | - | - |
| **AGMB01634** | - | - | - | **AGMB03213** | - | - | - | **AGMB00256** | - | - | - |
| **AGMB00919** | - | - | - | **AGMB01220** | - | - | - | **AGMB00481** | - | - | - |
| **AGMB00491** | - | - | - | **AGMB01961** | - | - | - | **AGMB00188** | - | - | - |
| **AGMB00489** | - | - | - | **AGMB01073** | - | - | - | **AGMB00197** | - | - | - |
| **AGMB01122** | - | - | - | **AGMB02841** | - | - | - | **AGMB00508** | - | - | - |
| **AGMB01124** | - | - | - | **AGMB01859** | - | - | - | **AGMB00464** | - | - | - |
| **AGMB00553** | - | - | - | **AGMB02831** | - | - | - | **AGMB00927** | - | - | - |
| **AGMB01689** | - | - | - | **AGMB01718** | - | - | - | **AGMB00604** | - | - | - |
| **AGMB00792** | - | - | - | **AGMB01632** | - | - | - | **AGMB01100** | - | - | - |
| **AGMB01314** | - | - | - | **AGMB00825** | - | - | - | **AGMB00519** | - | - | - |
| **AGMB00891** | - | - | - | **AGMB01728** | - | - | - | **AGMB00926** | - | - | - |
| **AGMB00631** | - | - | - | **AGMB01846** | - | - | - | **AGMB00963** | - | - | - |
| **AGMB00124** | - | - | - | **AGMB01880** | - | - | - | **AGMB00548** | - | - | - |
| **AGMB01585** | - | - | - | **AGMB01306** | - | - | - | **AGMB01139** | - | - | - |
| **AGMB01110** | - | - | - | **AGMB01316** | - | - | - | **AGMB00592** | - | - | - |
| **AGMB00378** | - | - | - | **AGMB02179** | - | - | - | **AGMB00820** | - | - | - |
| **AGMB00271** | - | - | - | **AGMB01706** | - | - | - | **AGMB00912** | **+** | **+** | **+** |
| **AGMB00230** | - | - | - | **AGMB02332** | - | - | - | **AGMB00490** | - | - | - |
| **AGMB00771** | - | - | - | **AGMB02211** | - | - | - | **AGMB00274** | - | - | - |
| **AGMB02829** | - | - | - | **AGMB02029** | - | - | - | **AGMB00905** | - | - | - |
| **AGMB03006** | - | - | - | **AGMB01105** | - | - | - | **AGMB01083** | - | - | - |
| **AGMB01580** | - | - | - | **AGMB00979** | - | - | - | **AGMB00357** | - | - | - |
| **AGMB02990** | - | - | - | **AGMB00829** | - | - | - | **AGMB01135** | - | - | - |
| **AGMB03219** | - | - | - | **AGMB01121** | - | - | - | **AGMB00181** | - | - | - |
| **AGMB01659** | - | - | - | **AGMB00544** | - | - | - | **AGMB00259** | - | - | - |
| **AGMB00170** | - | - | - | **AGMB00108** | - | - | - | **AGMB00462** | - | - | - |
| **AGMB00869** | - | - | - | **AGMB00609** | - | - | - |  |  |  |  |
| **AGMB02774** | - | - | - | **AGMB01106** | - | - | - |  |  |  |  |

^a^ Antimicrobial activity: -; No inhibition, +; Zone of inhibition > 10 mm.

CFS, cell-free supernatant; ETEC, enterotoxigenic *Escherichia coli.*

**Supplementary Table 4.** Predicted functional genes associated with organic acid production in the *L. argentoratensis* AGMB00912 genome

| **EC No.** | **Product** | **Locus tag** |
| --- | --- | --- |
| **Both homo- and heterofermentation** | | |
| 2.7.1.2 | Glucokinase | AGMB00912_01350 |
| 1.2.1.12 | Glyceraldehyde-3-phosphate dehydrogenase | AGMB00912_00650 |
| 2.7.2.3 | Phosphoglycerate kinas | AGMB00912_00651 |
| 5.4.2.11 | Phosphoglycerate mutase | AGMB00912_00533 |
|  |  | AGMB00912_00747 |
|  |  | AGMB00912_02620 |
| 4.2.1.11 | Phosphopyruvate hydratase | AGMB00912_00653 |
| 2.7.1.40 | Pyruvate kinase | AGMB00912_01628 |
| **Homofermentation only** | | |
| 5.3.1.9 | Glucose-6-phosphate isomerase | AGMB00912_02084 |
| 2.7.1.11 | 6-phosphofructokinase | AGMB00912_01629 |
| 4.1.2.13 | Fructose-bisphosphate aldolase | AGMB00912_00285 |
| 5.3.1.1 | Triose-phosphate isomerase | AGMB00912_00652 |
| **Heterofermentation only** | | |
| 1.1.1.49 | Glucose-6-phosphate dehydrogenase | AGMB00912_02222 |
| 3.1.1.31 | 6-phosphogluconolactonase | AGMB00912_01885 |
|  |  | AGMB00912_01886 |
| 1.1.1.44 | Phosphogluconate dehydrogenase | AGMB00912_01020 |
| 1.1.1.343 | Phosphogluconate dehydrogenase | AGMB00912_01318 |
| 5.1.3.1 | Ribulose-phosphate 3-epimerase | AGMB00912_01400 |
| 4.1.2.9 | Phosphoketolase | AGMB00912_02204 |
|  |  | AGMB00912_02908 |
| 2.3.1.8 | Phosphate acetyltransferase | AGMB00912_00666 |
| 1.2.1.10 | Acetaldehyde dehydrogenase | AGMB00912_00284 |
|  |  | AGMB00912_02931 |
| 1.1.1.1 | Alcohol dehydrogenase | AGMB00912_01436 |
| **Lactate production** | | |
| 4.2.1.32 | L-lactate dehydrogenase | AGMB00912_00415 |
|  |  | AGMB00912_00920 |
| 1.1.1.28 | D-lactate dehydrogenase | AGMB00912_00727 |
|  |  | AGMB00912_01746 |
| 5.1.2.1 | Lactate racemase | AGMB00912_00092 |
| 1.1.1.38 | Malate dehydrogenase | AGMB00912_00923 |
| 1.1.1.101 | (S)-malate carboxylase | AGMB00912_00934 |
| **Fumarate production** | | |
| 1.1.1.38 | Malate dehydrogenase | AGMB00912_00923 |
| 4.2.1.2 | Fumarate hydratase (class II) | AGMB00912_00928 |
| **Acetate production** | | |
| 2.7.2.1 | Acetate kinase | AGMB00912_00185 |
|  |  | AGMB00912_00264 |
|  |  | AGMB00912_01933 |
| 1.2.3.3 | Pyruvate oxidase | AGMB00912_00705 |
|  |  | AGMB00912_00707 |
|  |  | AGMB00912_02184 |
| 2.3.1.8 | Phosphate acetyltransferase | AGMB00912_00666 |
| **Formate production** | | |
| 2.3.1.54 | Formate acetyltransferase | AGMB00912_02748 |

**Supplementary Table 5.** Representative characteristics of *L. argentoratensis*

**Supplementary Table 6.** MIC of antibiotics to inhibits the growth of AGMB00912.

| Antibiotics | Cut-off value (mg/L)^a^ | MIC (mg/L) |
| --- | --- | --- |
| Ampicillin | 4 | 0.125 |
| Gentamycin | 16 | 4 |
| Kanamycin | 64 | 48 |
| Streptomycin | 64 | 64 |
| Erythromycin | 1 | 0.25 |
| Chloramphenicol | 4 | 48 |
| Tetracycline | 8 | 1.5 |
| Clindamycin | 4 | 0.125 |

^a^Cut-off value which was recommended by EFSA in 2018

**Supplementary Table 7.** 16S rRNA gene analysis preprocessing and quality control of ASV data.

| Group | | Name | | Total bases (bp) | | Total reads | | | | GC (%) | | AT (%) | | Q20 (%) | | | Q30 (%) | |
| --- | --- | --- | --- | --- | --- | --- | --- | --- | --- | --- | --- | --- | --- | --- | --- | --- | --- | --- |
| Control | | Con_1 | | 44,980,236 | | 149,436 | | | | 52.1 | | 47.9 | | 90.4 | | | 81.6 | |
|  |  | Con_2 | | 49,935,900 | | 165,900 | | | | 52.5 | | 47.5 | | 88.8 | | | 79.3 | |
|  |  | Con_3 | | 41,757,730 | | 138,730 | | | | 51.0 | | 49.0 | | 91.1 | | | 82.5 | |
|  |  | Con_4 | | 41,977,460 | | 139,460 | | | | 54.1 | | 45.9 | | 89.1 | | | 79.5 | |
|  |  | Con_5 | | 42,540,330 | | 141,330 | | | | 50.6 | | 49.4 | | 91.0 | | | 82.3 | |
| LA | | LA_1 | | 48,512,772 | | 161,172 | | | | 53.7 | | 46.3 | | 90.1 | | | 81.1 | |
|  |  | LA_2 | | 44,343,922 | | 147,322 | | | | 52.9 | | 47.1 | | 90.3 | | | 81.4 | |
|  |  | LA_3 | | 51,841,832 | | 172,232 | | | | 53.5 | | 46.5 | | 89.8 | | | 80.7 | |
|  |  | LA_4 | | 57,726,984 | | 191,784 | | | | 52.1 | | 47.9 | | 90.2 | | | 81.2 | |
|  |  | LA_5 | | 49,502,460 | | 164,460 | | | | 52.7 | | 47.3 | | 90.4 | | | 81.4 | |
| EC | | EC_1 | | 39,730,796 | | 131,996 | | | | 51.8 | | 48.2 | | 90.1 | | | 81.0 | |
|  |  | EC_2 | | 48,321,336 | | 160,536 | | | | 51.8 | | 48.2 | | 90.8 | | | 82.0 | |
|  |  | EC_3 | | 45,776,080 | | 152,080 | | | | 51.8 | | 48.2 | | 90.4 | | | 81.8 | |
|  |  | EC_4 | | 48,695,780 | | 161,780 | | | | 52.2 | | 47.8 | | 88.1 | | | 78.6 | |
|  |  | EC_5 | | 43,818,376 | | 145,576 | | | | 50.9 | | 49.1 | | 91.2 | | | 82.7 | |
| LE | | LE_1 | | 43,838,242 | | 145,642 | | | | 50.7 | | 49.3 | | 90.7 | | | 81.7 | |
|  |  | LE_2 | | 55,781,922 | | 185,322 | | | | 53.3 | | 46.7 | | 90.6 | | | 81.8 | |
|  |  | LE_3 | | 52,631,054 | | 174,854 | | | | 54.1 | | 45.9 | | 90.0 | | | 80.9 | |
|  |  | LE_4 | | 43,518,580 | | 144,580 | | | | 52.1 | | 47.9 | | 89.5 | | | 80.1 | |
|  |  | LE_5 | | 57,056,356 | | 189,556 | | | | 52.5 | | 47.5 | | 90.2 | | | 81.4 | |
|  | | | | | | | | | | | | | | | | | | |
| **Data processing** | | | | | | | | | | | | | | | | | | |
| Group | Name | | Raw data | Adapter & Primer Trimming | Preprocessing Length Trimming | | Quality Filter | QC Remain | denoisedFor | | denoisedRev | | mergedPair | | non-chimeric | ASV Length Filter | | ASV Remain |
| Control | Con_1 | | 74,718 | 73,210 | 73,210 | | 64,697 | 86.59% | 63,002 | | 63,781 | | 56,700 | | 33,201 | 33,201 | | 44.44% |
|  | Con_2 | | 82,950 | 81,108 | 81,108 | | 68,981 | 83.16% | 66,014 | | 67,691 | | 55,108 | | 41,914 | 41,914 | | 50.53% |
|  | Con_3 | | 69,365 | 67,988 | 67,988 | | 60,600 | 87.36% | 59,161 | | 59,808 | | 53,945 | | 36,408 | 36,408 | | 52.49% |
|  | Con_4 | | 69,730 | 68,351 | 68,351 | | 58,631 | 84.08% | 56,680 | | 57,367 | | 46,777 | | 29,046 | 29,046 | | 41.65% |
|  | Con_5 | | 70,665 | 69,225 | 69,225 | | 61,638 | 87.23% | 60,321 | | 60,770 | | 55,692 | | 39,500 | 39,500 | | 55.90% |
| LA | LA_1 | | 80,586 | 78,965 | 78,965 | | 69,011 | 85.64% | 66,747 | | 67,897 | | 57,360 | | 33,268 | 33,268 | | 41.28% |
|  | LA_2 | | 73,661 | 72,025 | 72,025 | | 63,221 | 85.83% | 61,897 | | 62,589 | | 56,285 | | 35,861 | 35,861 | | 48.68% |
|  | LA_3 | | 86,116 | 84,487 | 84,487 | | 73,261 | 85.07% | 70,124 | | 71,815 | | 58,300 | | 37,599 | 37,599 | | 43.66% |
|  | LA_4 | | 95,892 | 94,104 | 94,104 | | 82,494 | 86.03% | 78,733 | | 80,689 | | 65,154 | | 48,629 | 48,629 | | 50.71% |
|  | LA_5 | | 82,230 | 80,614 | 80,614 | | 71,134 | 86.51% | 69,331 | | 70,114 | | 60,236 | | 37,833 | 37,833 | | 46.01% |
| EC | EC_1 | | 80,268 | 78,644 | 78,644 | | 69,609 | 86.72% | 68,037 | | 68,539 | | 62,607 | | 31,891 | 31,891 | | 39.73% |
|  | EC_2 | | 76,040 | 74,511 | 74,511 | | 65,754 | 86.47% | 64,197 | | 64,966 | | 60,354 | | 48,040 | 48,040 | | 63.18% |
|  | EC_3 | | 80,890 | 78,883 | 78,883 | | 65,659 | 81.17% | 63,282 | | 64,627 | | 54,092 | | 32,433 | 32,433 | | 40.10% |
|  | EC_4 | | 72,788 | 71,274 | 71,274 | | 63,908 | 87.80% | 63,118 | | 63,395 | | 59,986 | | 39,957 | 39,954 | | 54.89% |
|  | EC_5 | | 65,998 | 64,747 | 64,747 | | 56,560 | 85.70% | 54,792 | | 55,608 | | 48,770 | | 34,243 | 34,241 | | 51.88% |
| LE | LE_1 | | 72,821 | 71,326 | 71,326 | | 63,009 | 86.53% | 61,159 | | 62,068 | | 55,277 | | 42,312 | 42,312 | | 58.10% |
|  | LE_2 | | 92,661 | 90,920 | 90,920 | | 80,319 | 86.68% | 77,346 | | 78,427 | | 64,354 | | 41,871 | 41,871 | | 45.19% |
|  | LE_3 | | 87,427 | 85,725 | 85,725 | | 75,446 | 86.30% | 73,143 | | 74,193 | | 61,275 | | 36,902 | 36,902 | | 42.21% |
|  | LE_4 | | 72,290 | 70,641 | 70,641 | | 61,156 | 84.60% | 59,821 | | 60,478 | | 54,250 | | 37,218 | 37,218 | | 51.48% |
|  | LE_5 | | 94,778 | 92,823 | 92,823 | | 81,821 | 86.33% | 78,998 | | 80,466 | | 68,550 | | 51,844 | 51,844 | | 54.70% |

**Supplementary Figure 1.** Inhibition zones of pathogen strains (ETEC K88ac, ETEC K99, STEC, and ST454) treated with cell-free supernatant of AGMB00912.


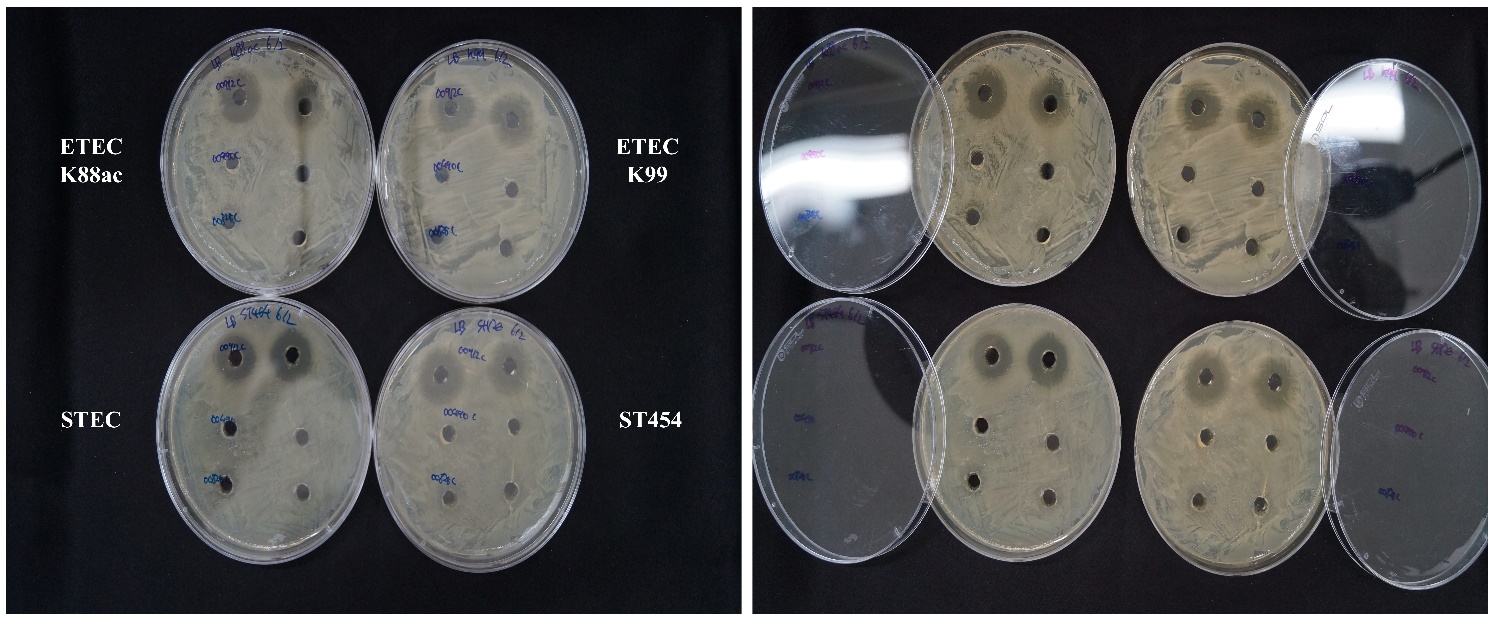


**Supplementary Figure 2.** Growth of AGMB00912 and enterotoxigenic *Escherichia coli* (ETEC K88ac, K88ab, K99), Shiga toxin-producing *Escherichia coli* (STEC Stx2e, STa/LT/Stx2e, STa/987p), *Salmonella* Typhimurium (ST454) in a co-culture MRS medium.


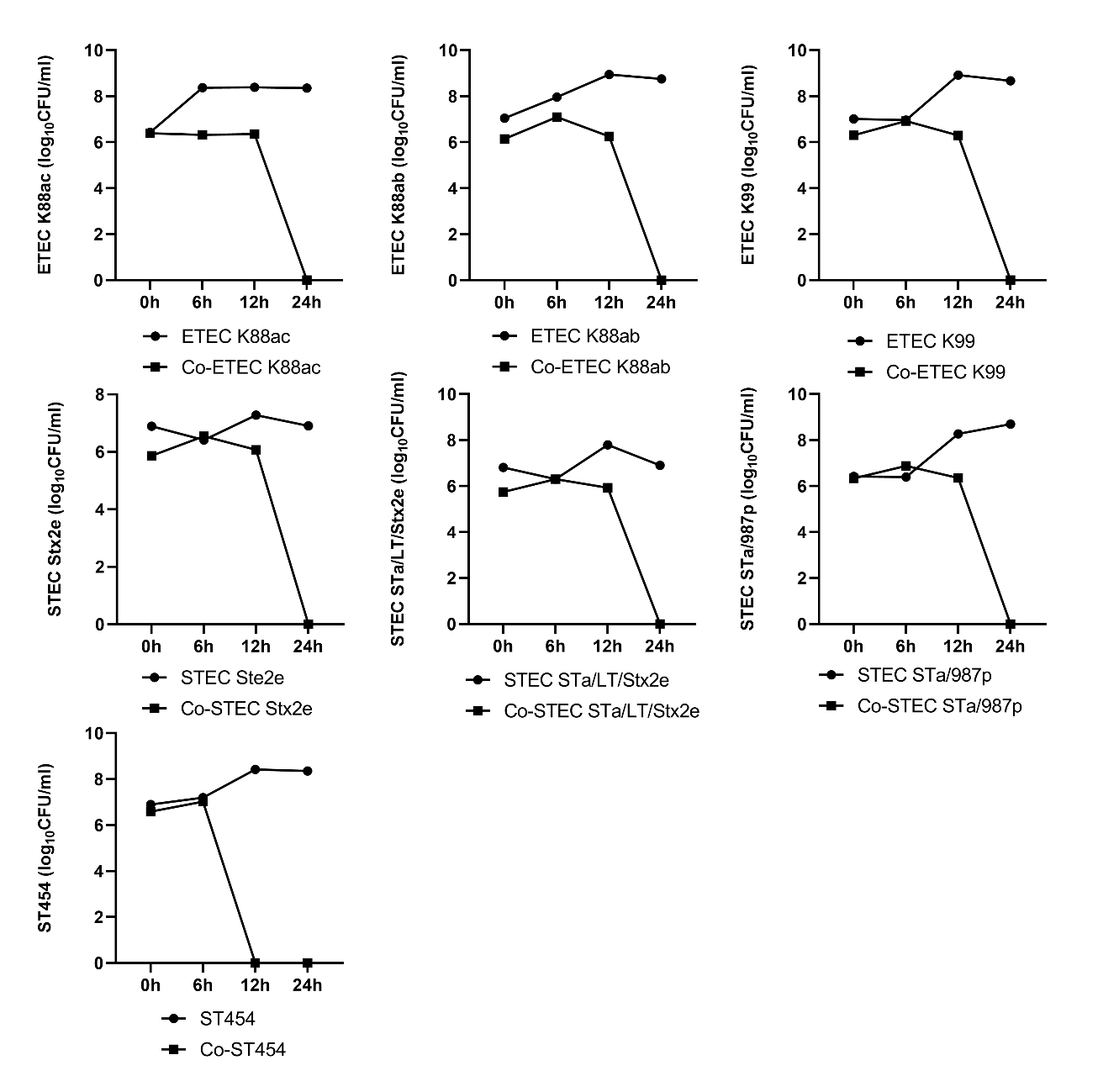


**Supplementary Figure 3.** Average nucleotide analysis of *Lactiplantibacillus* spp. including *L. argentoratensis* AGMB00912 using a phylogenetic tree. The phylogenetic tree was generated based on distance values. The scale bar represents 0.03 of the distance value, which means a 3% difference in genetic comparison. Red text indicates *L. argentoratensis* AGMB00912. The tree contains four clusters, namely, Cluster I, II, III, and IV.

**
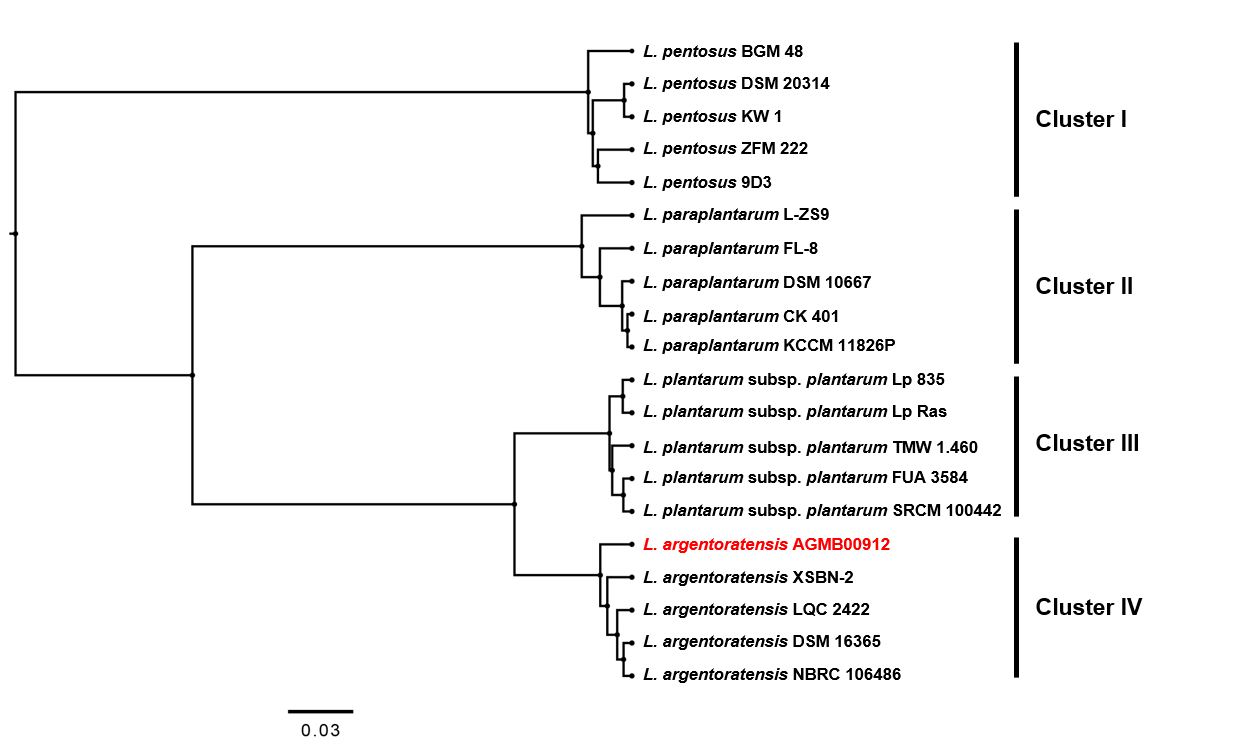
****Supplementary Figure 4.** Safety evaluation of AGMB00912. (A) Cytotoxicity to Caco-2, a human colon carcinoma cell line. *E. coli* O157:H7 ATCC 43895 was used as the cytotoxic control strain.The negative control means the group which was treated by basal media. (B-E) Hemolytic activity of (B) *Staphylococcus salivarius* ATCC 19250, (C) *S. aureus* ATCC 29213, (D) *S. epidermis* ATCC and (E) AGMB00912. Statistical analysis was conducted using ordinary one-way ANOVA with Dunnett correction, comparing to negative control (NC). **, *P*<0.01; ***, *P*<0.001.


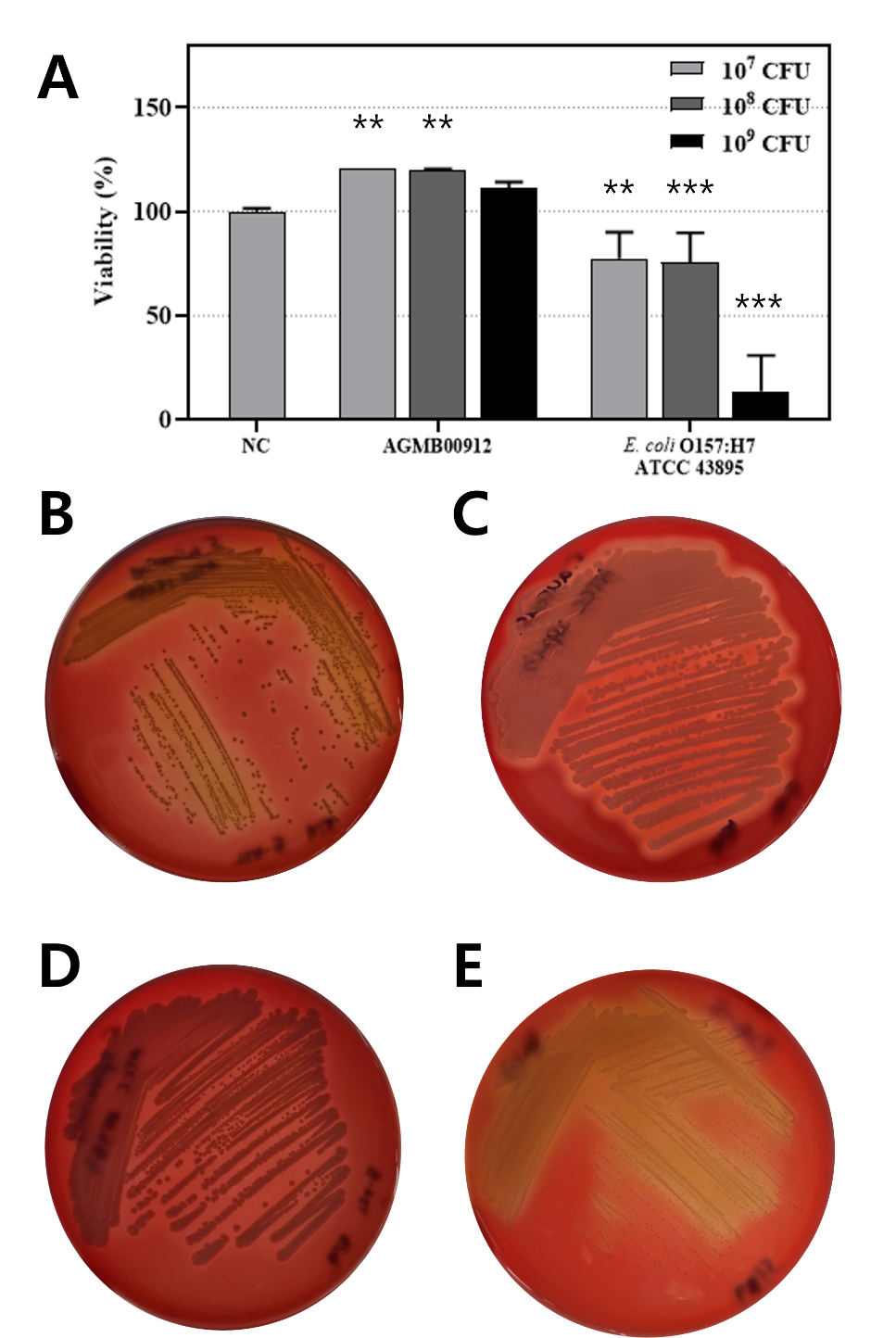


**Supplementary Figure 5.** Core microbiome in different groups of mice. Heatmap depicting the core operational taxonomic units (OTUs) and their prevalence at different detection thresholds for (A) Con, (B) EC, (C) LA, (D) LE.

**
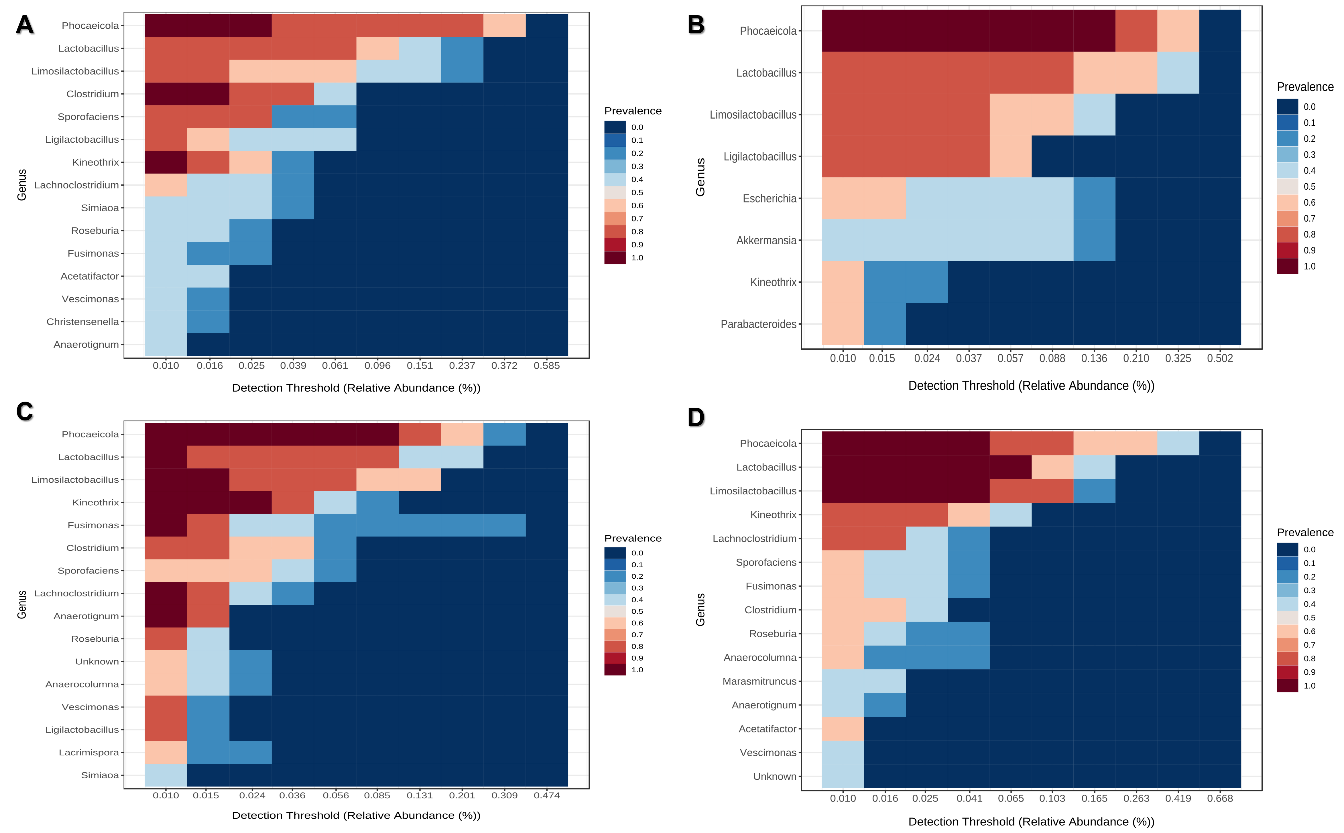
**

**Supplementary Figure 6.** The substantial differences in metabolic functions among the EC, LA, and LE groups includes 28 pathways.


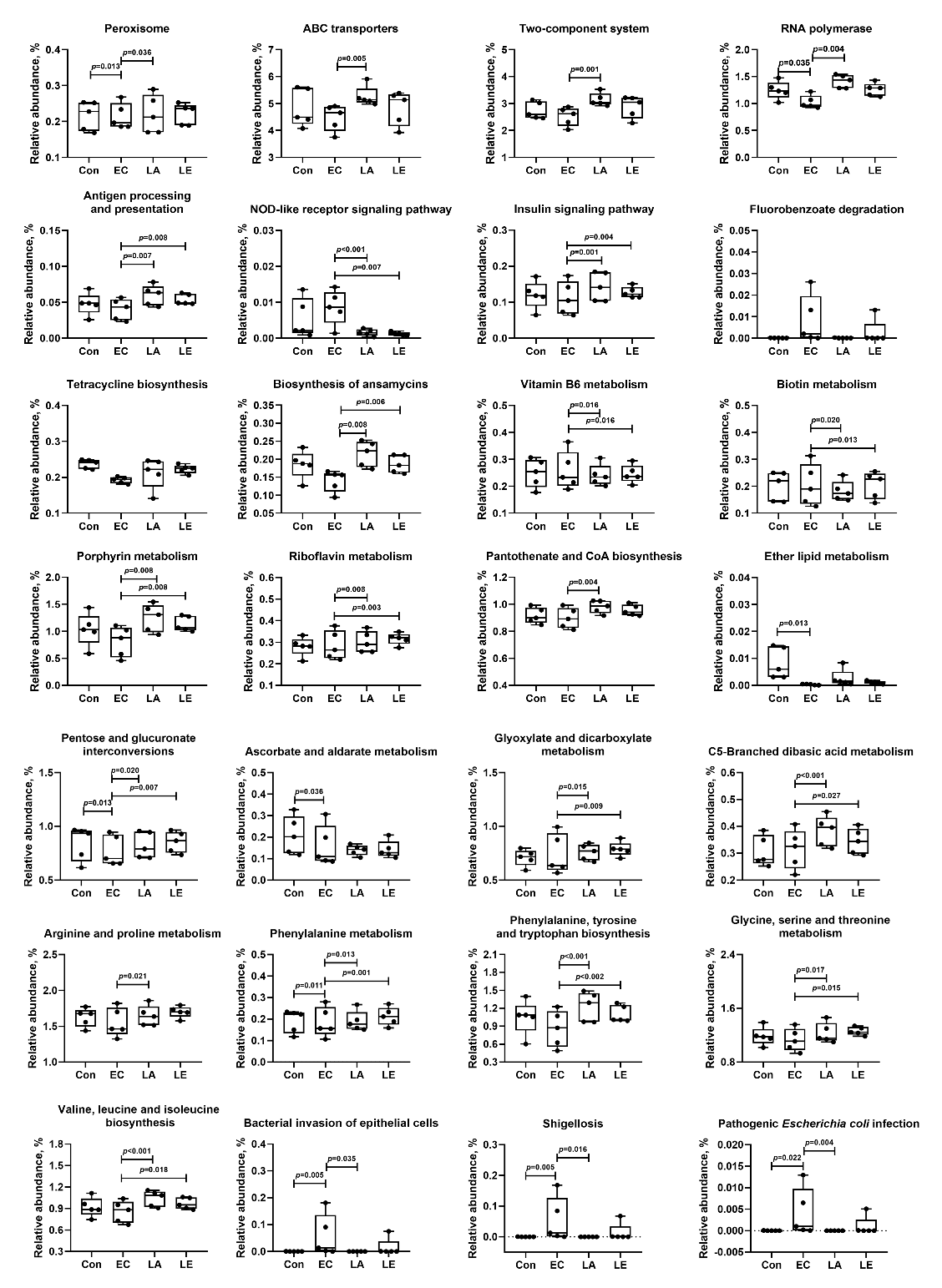

Supplement: Supplementary file 2 [file Table_2.DOCX]
