# Supplementary figures and images for: Lactiplantibacillus argentoratensis AGMB00912 protects weaning mice from ETEC infection and enhances gut health
Source: Front Microbiol. 2024 Sep 10;15:1440134. doi: 10.3389/fmicb.2024.1440134 (PMC11420142; doi:10.3389/fmicb.2024.1440134)

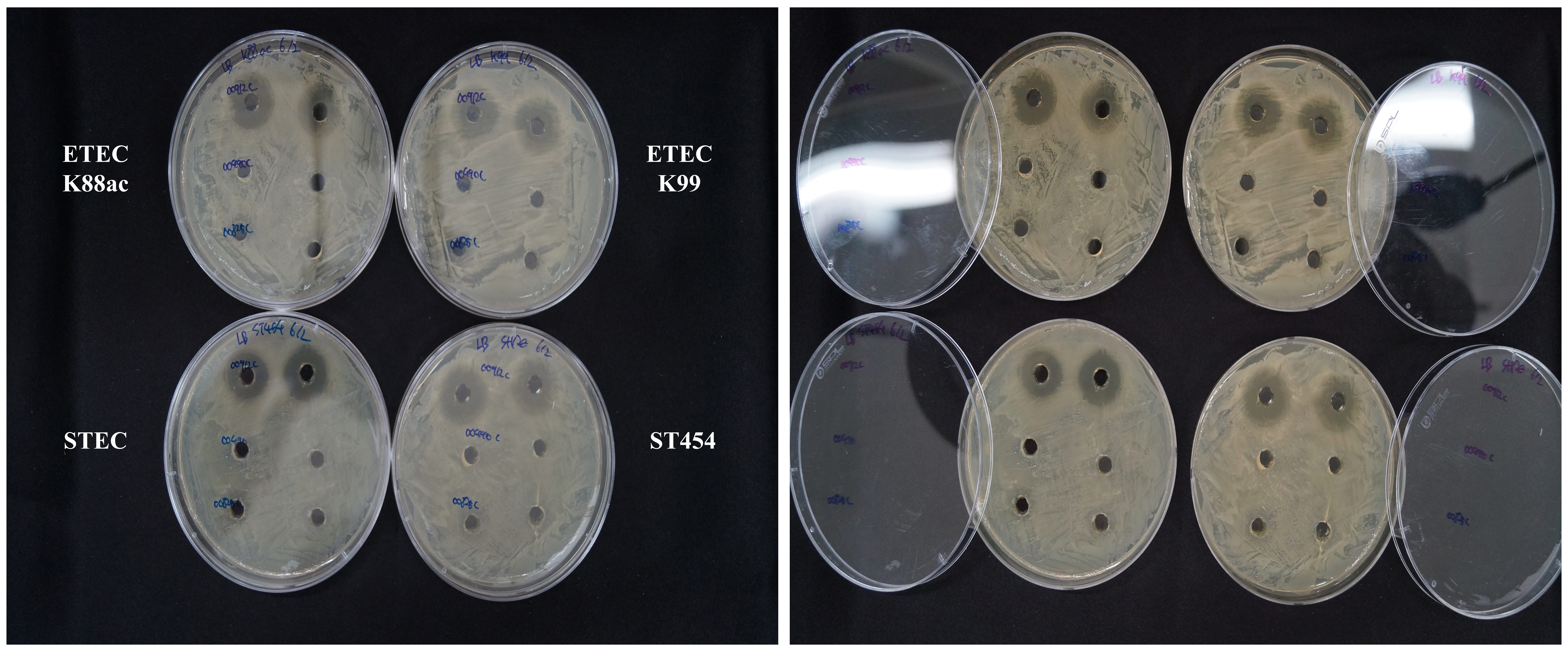

Supplement: Supplementary file 3 [file Image_1.TIF]

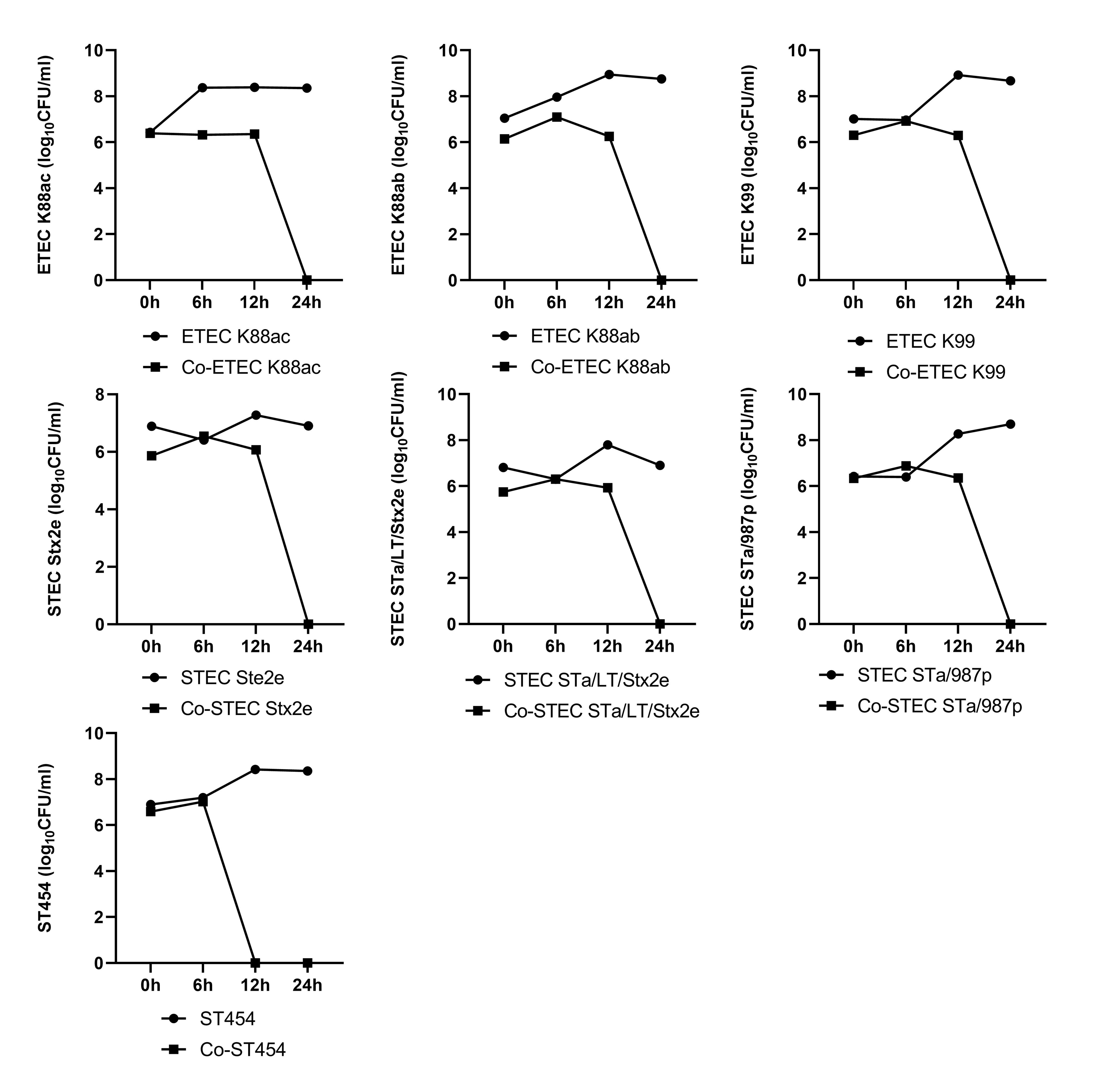

Supplement: Supplementary file 4 [file Image_2.TIF]

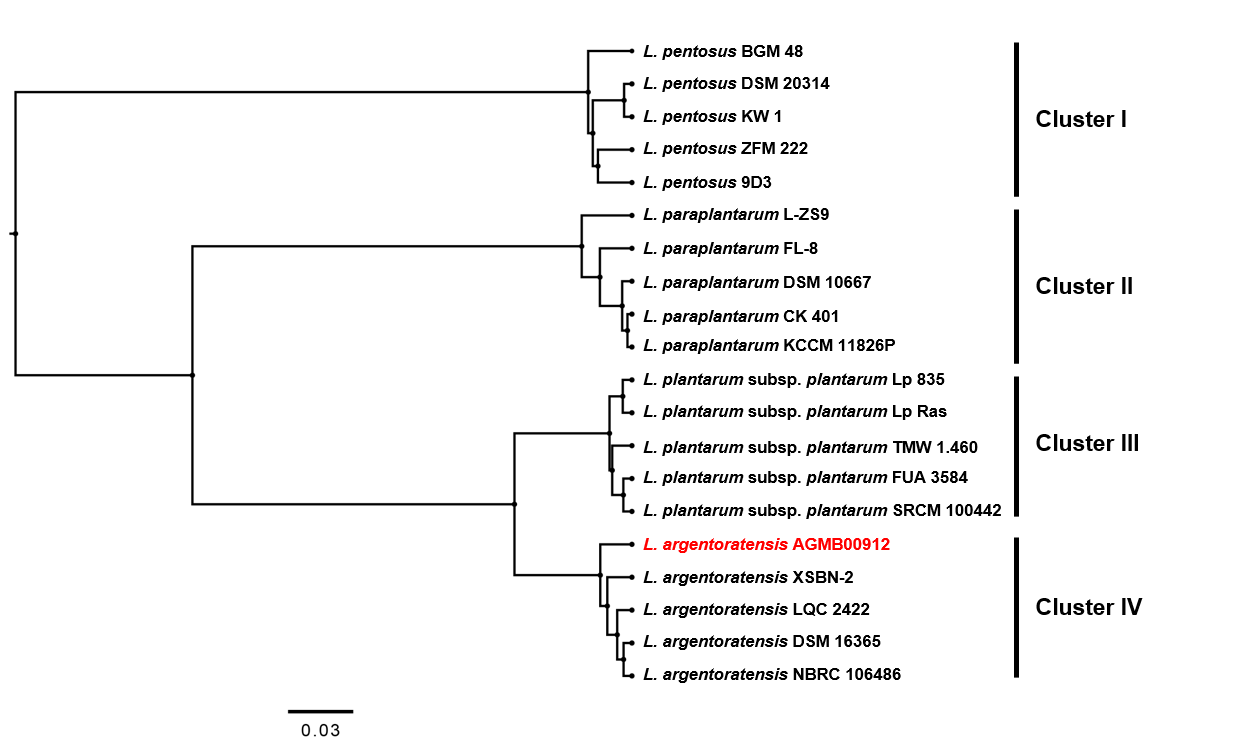

Supplement: Supplementary file 5 [file Image_3.TIF]

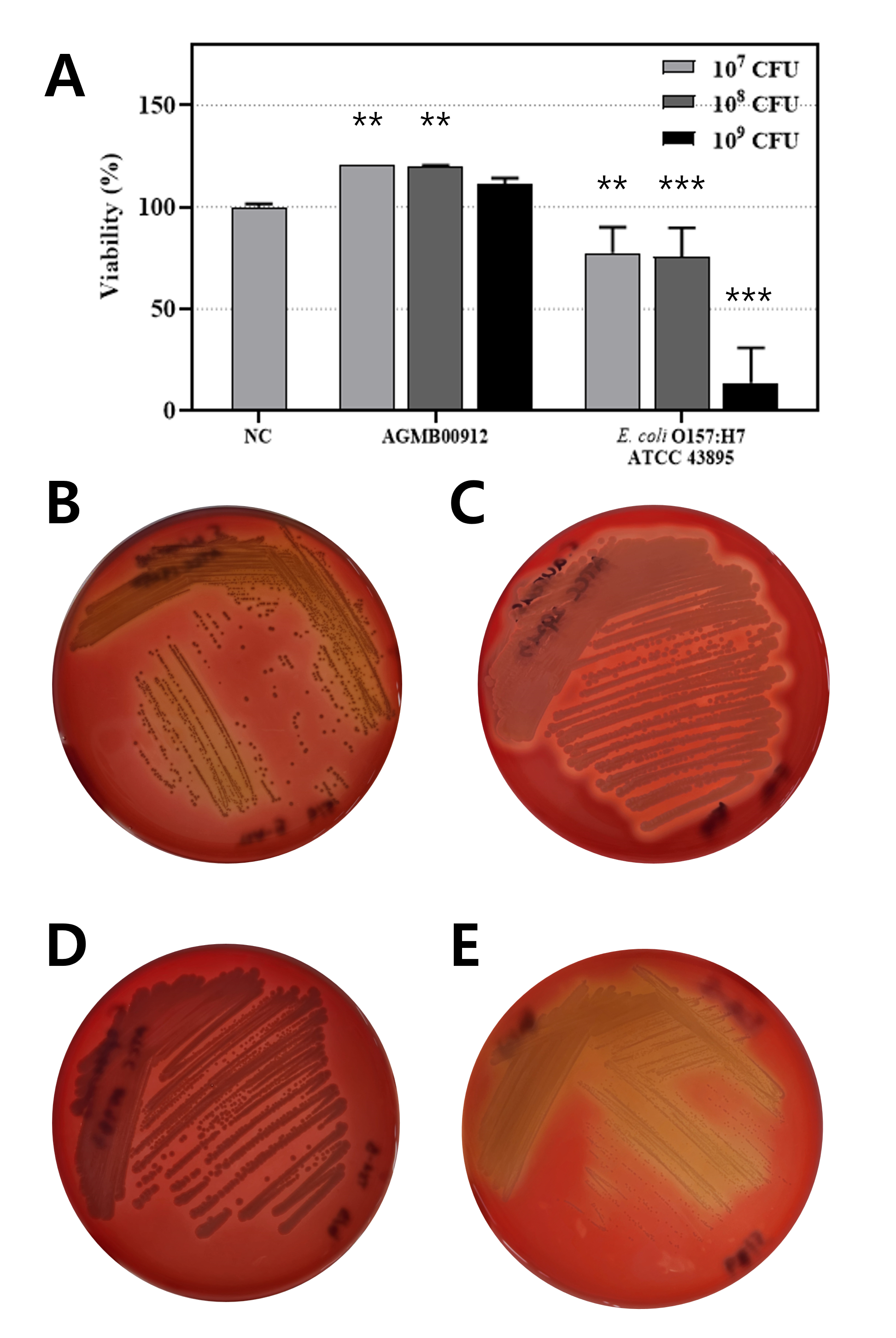

Supplement: Supplementary file 6 [file Image_4.TIF]

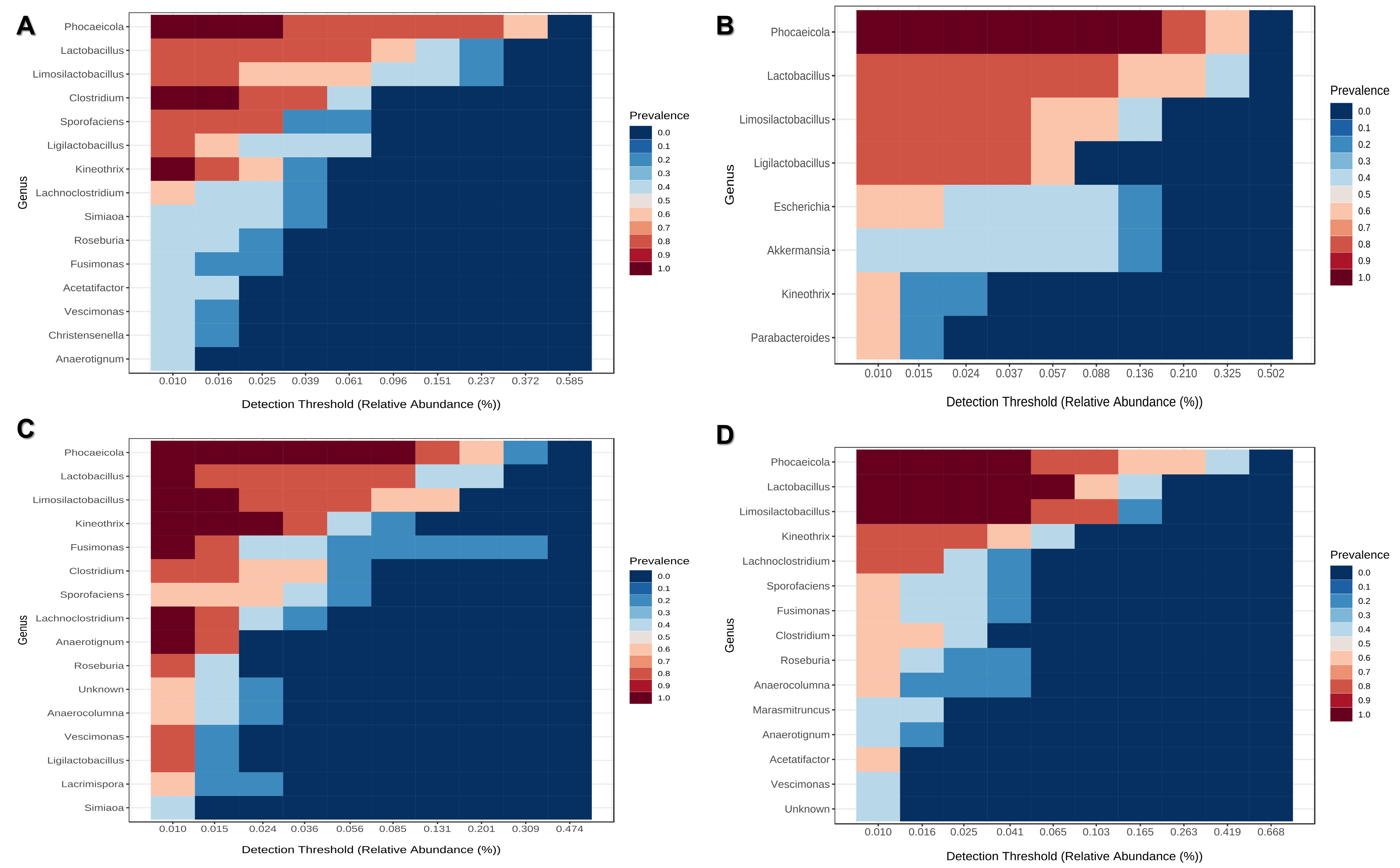

Supplement: Supplementary file 7 [file Image_5.TIF]

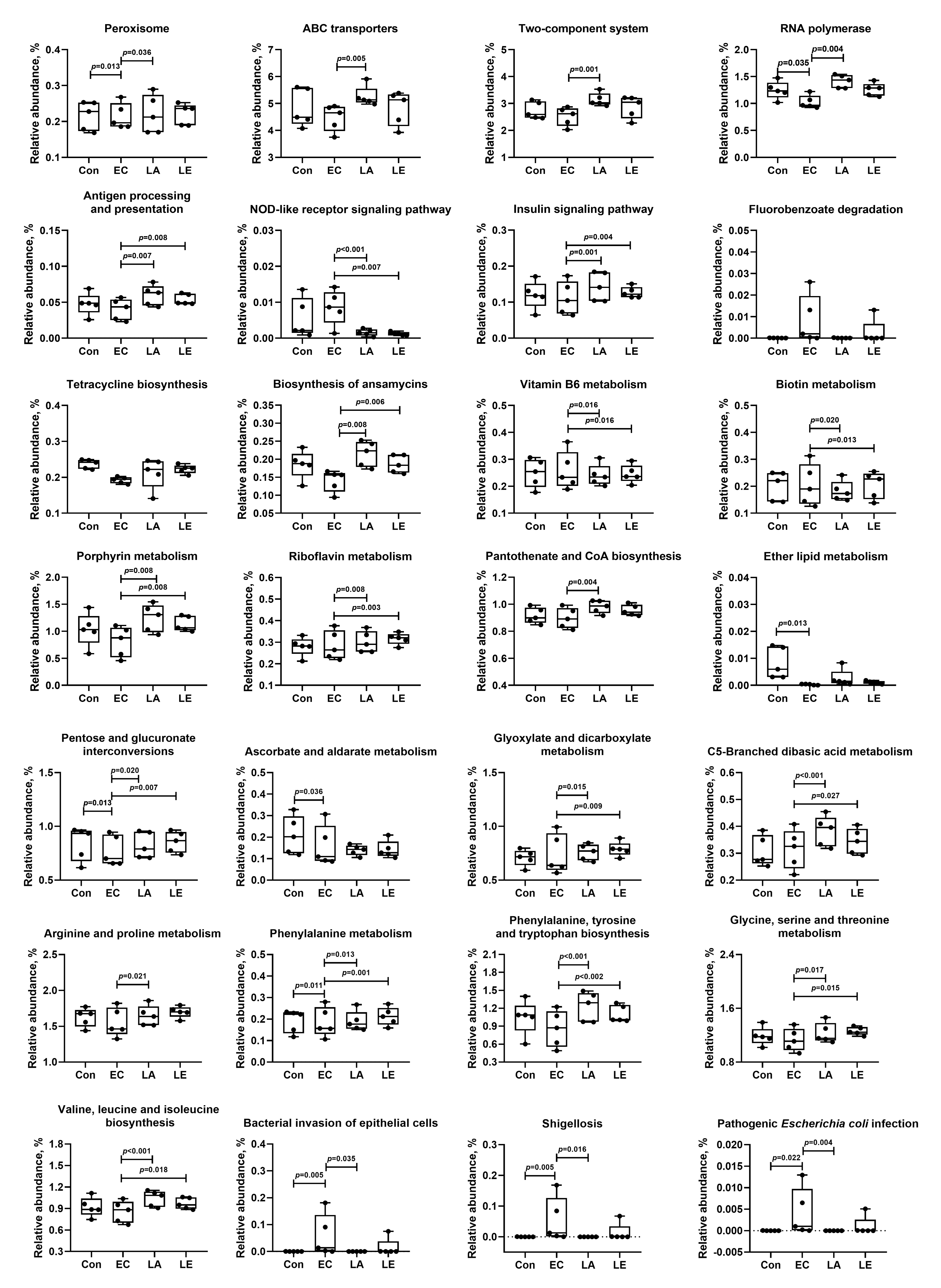

Supplement: Supplementary file 8 [file Image_6.TIF]
